# Efficacy, safety, and tolerability of albendazole and ivermectin based regimens for the treatment of microfilaraemic loiasis in adult patients in Gabon: A randomized controlled assessor blinded clinical trial

**DOI:** 10.1371/journal.pntd.0011584

**Published:** 2023-08-28

**Authors:** Rella Zoleko-Manego, Ruth Kreuzmair, Luzia Veletzky, Wilfrid Ndzebe-Ndoumba, Dorothea Ekoka Mbassi, Dearie G. Okwu, Lia B. Dimessa-Mbadinga-Weyat, Roselyne D. Houtsa-Temgoua, Johannes Mischlinger, Matthew B. B. McCall, Peter G. Kresmner, Selidji T. Agnandji, Betrand Lell, Ayôla A. Adegnika, Ghyslain Mombo-Ngoma, Michael Ramharter

**Affiliations:** 1 Centre de Recherches Médicales de Lambaréné, Lambaréné, Gabon; 2 Department of Tropical Medicine, Bernhard Nocht Institute for Tropical Medicine & I. Department of Medicine, University Medical Center Hamburg-Eppendorf, Hamburg, Germany; 3 German Center for Infection Research–Partner Sites Hamburg-Lübeck-Borstel-Riems, Germany; 4 Radboud University Medical Center, Nijmegen, Netherlands; 5 Institute of Tropical Medicine, University of Tübingen, Tübingen, Germany; 6 German Center for Infection Research–Partner Site Tübingen, Germany; 7 Division of Infectious Diseases and Tropical Medicine, Department of Medicine 1, Medical University of Vienna, Vienna, Austria; 8 Department of Implementation Research, Bernhard Nocht Institute for Tropical Medicine & I. Department of Medicine, University Medical Center Hamburg-Eppendorf, Hamburg, Germany; University of Agricultural Sciences and Veterinary Medicine Cluj-Napoca, Life Science Institute, ROMANIA

## Abstract

**Background:**

There is a lack of systematic evidence for strategies to control loiasis transmission in highly endemic regions. Here we assessed albendazole and ivermectin based treatment regimens to reduce *Loa loa* microfilaraemia in Gabon.

**Methods:**

Eligible adult patients with *L*. *loa* microfilaraemia between 5,000 and 50,000 microfilariae/ml were randomized to either a control or one of three intervention groups (1:2:2:2 allocation ratio) consisting of three-week twice daily 400mg oral albendazole followed by 1) no treatment, 2) two further weeks of twice daily 400mg albendazole, or 3) a single dose of ivermectin in this open label randomized assessor blinded controlled clinical trial. The primary outcome was the proportion of participants with *L*. *loa* microfilaraemia ≤ 100 mf/ml at Day 168.

**Results:**

In the efficacy-population of 42 patients 0 (0%; control group), 1 (9%; 3-week albendazole), 5 (39%; 5-weeks albendazole) and 2 (22%; 3-week albendazole plus single dose ivermectin) participants met the primary outcome of microfilaraemia below 100/ml at day 168. A 80–90% reduction of microfilaraemia was observed in the active treatment groups.

**Conclusion:**

The 5-week regimen of albendazole or a 3-week regimen of albendazole followed by ivermectin were most efficacious to reduce microfilaraemia. All therapeutic regimens were well tolerated and safe.

**Trial registration:**

Trial registered at the Pan-African Clinical Trials Registry: PACTR201807197019027.

## Author summary

To date there is a lack of safe treatment options to be used for the control of *Loa loa* transmission in highly endemic regions. This study assessed the parasitological efficacy of albendazole alone (three or five weeks regimen) or albendazole (three weeks regimen) with a single dose of ivermectin for the reduction of microfilaraemia in adult patients in Gabon. This proof of concept study showed highest efficacy of about 90% reduction of microfilaraemia when albendazole was given twice daily for five weeks or albendazole was administered twice daily for three weeks followed by single dose ivermectin. As there were no safety concerns these findings indicate that albendazole based treatment regimens may in principle be useful for implementation in population-based control programs. To overcome the problem associated with prolonged oral treatment regimens new injectable drug formulations with extended release of active substances may be considered to facilitate implementation of such control programs.

## Introduction

Loiasis is a highly neglected infectious disease caused by the filarial nematode *Loa loa* typically manifesting as a migrating eye worm under the bulbar conjunctiva or transient peripheral oedemas. *L*. *loa* is endemic in West and Central Africa [1, 2] with more than 10 million persons infected and 30 million at risk of infection [3]. For a long time loiasis has been considered an infection with limited clinical importance easily tolerated by infected individuals in endemic regions [4, 5]. However, excess mortality in hyper-microfilaraemic individuals and substantial morbidity due to loiasis have been reported recently, thus highlighting the true burden of disease caused by loiasis in affected communities [6, 7]. Due to this underestimation of disease burden and its epidemiological overrepresentation in remote regions little attention was given to it by the scientific community and public health stakeholders [4].

Currently there are no control programs for this filarial disease in any of the endemic countries. To effectively reduce onwards transmission of this chronic infection, clearance of microfilaraemia is necessary to reduce infectiousness to the intermediate arthropod hosts *Chrysops silacea* and *C*. *dimidiata*. There is however a lack of evidence for appropriate drug regimens to be used to treat patients in endemic regions. Diethylcarbamazine (DEC) is known to be active against microfilariae and adult worms [4] but its use may lead to severe neurological complications with potentially fatal outcome in patients with high microfilarial load. In addition, DEC is contraindicated in patients harbouring the frequently co-endemic filarial pathogen *Onchocerca volvulus* when suffering from ocular onchocerciasis [8, 9].

Ivermectin is one of the most widely used antiparasitic drugs [10] and highly effective in reducing microfilaraemia of *L*. *loa*. Its use in highly microfilaraemic individuals (> 30,000 microfilariae per millilitre of blood, mf/mL) was shown to be associated with the risk for severe neurological adverse reactions with potentially fatal outcome [5, 7]. It was later shown that the risk for severe adverse drug reactions is closely linked to the quantity of peripheral microfilaraemia with substantial risk in individuals harbouring microfilareamia ≥ 8,000 mf/mL [7]. Ivermectin can therefore only be safely administered to loiasis patients with proven low microfilaraemia [11] and is therefore not suitable for the indiscriminate use as in mass drug administration programs in loiasis endemic regions.

Albendazole has been demonstrated to exert a slow but reliable activity against microfilariae of *L*. *loa* and is perceived to gradually kill adult worms. Due to its rather slow onset of action, it is commonly used over a 21 day period to safely reduce *L*. *loa* microfilaraemia before initiation of treatment with a rapidly acting anthelminthic drug [12–15].

The clinical evaluation of treatment approaches suitable for mass drug administration programs are necessary to ensure that these regimens prove safe and effective in the control of loiasis. As DEC and ivermectin cannot be used without special precaution due to safety concerns in highly microfilaraemic individuals, the use of albendazole as initial drug constitutes currently the only viable treatment option to safely reduce microfilaraemia to a level that allows safe use of sequential therapy with another drug. The aim of this clinical trial was therefore to evaluate three alternative fixed treatment regimens for their efficacy to reduce microfilaraemia of *L*. *loa* infection in a highly endemic region in Gabon and to evaluate the safety and tolerability of the respective regimens.

## Patients and methods

### Ethics statement

The study was conducted according to the Declaration of Helsinki, the International Council for Harmonisation for Good Clinical Practice and the applicable laws and regulations in Gabon. The study was approved by the institutional ethics committee of the Centre de Recherches Médicales de Lambaréné (reference number: CEI-CERMEL, 010/2018). Study participants provided written informed consent before any study procedure was performed.

### Study design

This study was designed as a randomized controlled assessor-blinded clinical trial evaluating the efficacy and safety of three albendazole-based regimens versus a control group in adults with proven *L*. *loa* microfilaraemia in a highly endemic region in Gabon. The trial was conducted according to the study protocol (registered at the Pan-African Clinical Trials Registry: PACTR201807197019027) and no changes to the trial protocol or statistical analysis plan were made after trial commencement.

### Study *setting and population*

The study was conducted at the Centre de Recherches Médicales de Lambaréné (CERMEL), in the regions of Tsamba-Magotsi and Lambaréné, Gabon, from May 9 2018 to April 16 2020. The study region is highly endemic for *L*. *loa* and a range of other parasitic infectious diseases [6, 16, 17]. Residents of the study region participating in ongoing screening activities for blood borne filarial infections were invited to participate in this clinical trial. Participants aged above 18 years were screened based on the following inclusion criteria: presence of *L*. *loa* microfilaraemia between 5,000 and 50,000 mf/ml in peripheral blood during daytime sampling (10 a.m.– 3 p.m.) assessed microscopically by thick blood smears of capillary blood, residence in the study region, willingness to participate in this clinical trial for the entire 6-month follow-up period as evidenced by written informed consent. Exclusion criteria were reported intake of albendazole, ivermectin or DEC within 4 weeks prior to recruitment; intolerance or allergy to study drugs; pregnancy or lactation, known active viral hepatitis or other liver disease, known HIV infection or presence of an immunosuppressive disease, history of epilepsy, encephalitis, meningitis or encephalopathy, or any other acute or chronic medical condition judged by the investigator to put the potential participant at more than acceptable risk or to significantly affect the results of the study.

### Randomisation, enrolment and intervention

Eligible patients were randomized by an independent investigator to the inactive control group (CONTROL), or one of the following three albendazole-based fixed sequential treatment regimens: ALB, ALB-ALB, or ALB-IVM. Computer generated block randomization was used to generate the allocation list with a block size of 7 and an allocation ratio of 1:2:2:2. Once seven participants were judged eligible, the randomization procedure was initiated. Based on a sequential list of eligible patients, individuals dropping out before the end of the treatment period were replaced in the same treatment group with the next eligible participant. Participants in the control group (CONTROL) received symptomatic treatment with the antihistaminic drug loratadine 10 mg (Loratol Tabs. 10mg, Dafra Pharma GmbH) oral once daily, intake under supervision, for seven days. All participants enrolled in one of the three intervention groups received twice daily oral therapy with 400mg albendazole (Albendazole Tabs. 400mg, Medopharm, India) after food intake for 21 days. Supervision of drug intake was daily during the first week of treatment. For the remaining treatment duration directly observed treatment was done every second day and tablets for the unsupervised drug intakes were provided each time only for the following day. At each time point information on compliance and safety was provided. Participants randomized to the ALB-ALB group received an additional 14-day treatment course of twice daily 400 mg albendazole with the same supervision of drug intake as mentioned above. Participants allocated to the ALB-IVM group received supervised treatment with a single dose of 150 μg/kg ivermectin (Stromectol 3mg, MSD) on day 23. Ivermectin was administered only after assurance that *L*. *loa* microfilarial load was below 4000 mf/ml to minimize potential risks associated with the use of ivermectin in microfilaremic individuals. This conservative approach was taken to minimize study related risks. In case of microfilaraemia above this threshold, participants were transferred to the ALB + ALB treatment group.

### Clinical and safety assessments

Participants underwent a physical examination with demographic data and medical history recorded at the baseline visit. Blood samples were taken for haematology (red blood cell count, haemoglobin, haematocrit, leukocyte count with differential and platelet count), biochemistry (bilirubin, transaminases alanine aminotransferase and aspartate aminotransferase, urea, creatinine and glucose) and viral serology (hepatitis B and C). A urine pregnancy test (SAS Ultimate hCG—SA Scientific Ltd.) for women under the age of 55 was performed to rule out pregnancy and counselling to avoid conception during the study period was provided. Assessments of treatment adherence, clinical status, adverse events, and concomitant medications were recorded at each visit during the treatment and follow-up period. In addition, participants were advised to contact the study health centre in case of occurrence of any adverse event. Follow-up visits were conducted by a trained nurse and a fieldworker. Any potential signs or symptoms recorded by them were discussed with the study doctor for further assessment or necessary action. Tolerability and safety assessments were performed until study completion at Day-168 including the recording of adverse events and clinical laboratory test results. A further urine pregnancy test was done for female participants <55 years of age at the end of the study follow-up period. All participants randomized in the control group were offered a 28 days albendazole treatment regimen at the end of 6 months follow up period.

### Efficacy assessments

Two thick smears of 20μl venous or capillary blood were prepared at baseline, before study drug administration and on days 1, 3, 7, 10, 14, 17, 21, 23, 24, 26, 28, 30, 33, 35, 37, 42, 49, 56, 70, 84, 98, 112, 126, 140, 154 and 168. Blood smears were dried and stained with 20% Giemsa stain for 20 minutes according standard protocols [18]. Stained microscopic slides were examined at 10x objective magnification for detection and 100x objective magnification for species level identification by two microscopists qualified in the detection of microfilariae and unaware of treatment allocation. *Loa loa* microfilarial density was expressed as the number of microfilaria per ml of blood by multiplying the observed microfilariae in 20μl of blood with the factor 50 [19]. A blood smear was considered negative when no microfilarial parasite was detected in 20 μl of blood by any of the two microscopists. *Mansonella perstans* microfilaraemia, which is a common co-endemic filarial pathogen in this region [20], was assessed and recorded separately when present. Every effort has been made to collect blood samples between 10:00 a.m. and 3:00 p.m. to take into account the diurnal periodicity of filarial species. Blood was collected either by finger prick, or by venepuncture when blood collection for haematology and biochemistry was performed at the same time.

### Sample size calculation

This clinical trial was a proof-of-concept study without predefined statistical hypothesis testing between groups. A total of 42 patients were planned to be recruited to ensure adequate precision of the primary outcome, i.e. microfilarial density reduction, based on the natural variability of microfilaraemia. A total of 12 evaluable patients were planned for each of the three intervention groups, in addition to 6 patients in the control group, thus resulting in an overall treatment allocation ratio of 1:2:2:2. Assuming a 50% proportion of patients achieving a microfilaria reduction below 100/ml, this sample size allowed a precision of the point estimate with a 95% confidence interval ranging from 25–75%.

### Outcomes/Endpoints

Microfilarial density thresholds, below which onwards transmission by the Chrysops vector cannot be sustained, are currently unknown but may depend on several factors including the life span of the insect vector [21]. However, the determinants of transmission dynamics are the success rate of ingested microfilariae becoming infective larvae in an arthropod vector, and microfilaria density in the source of the human blood meal. Based on this current understanding the primary efficacy outcome was defined as the proportion of subjects reaching *L*. *loa* microfilarial density ≤100 mf/ml within 6 months.

Secondary efficacy outcomes were the proportion of individuals in each treatment group being amicrofilaraemic at the last observation on day 168; the median reduction of *L*. *loa* microfilaraemia at each month after onset of treatment and the median time from initiation of treatment to the individual nadir (lowest level of microfilaraemia) of *L*. *loa* microfilaraemia.

Principal safety outcomes were defined as the occurrence of any related or unrelated grade 3 adverse event or serious adverse event (SAE) and occurrence of all adverse events at least possibly related to study medication assessed after the initiation of study treatment. Adverse event type, severity, seriousness and relationship to study drugs were assessed by the investigator. Seriousness was determined based on the serious adverse event definition in the ICH guidelines.

### Statistical methods

**Fig 1:**
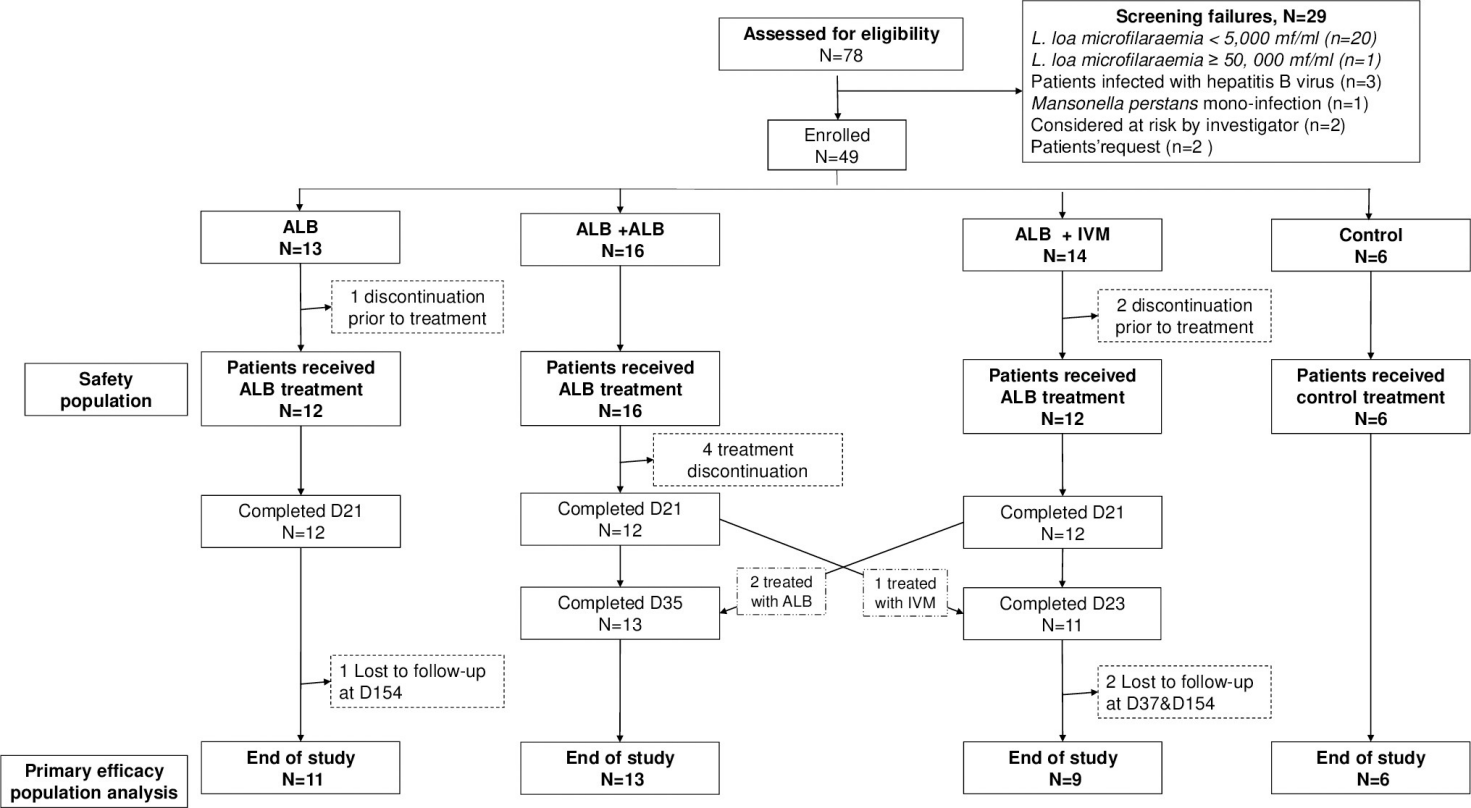
Study participants flow. Abbreviations: ALB: albendazole; IVM: ivermectin.

All data were collected on paper record forms that were entered into an electronic database (Research Electronic Data Capture, REDCap, Vanderbilt University) [22]. Statistical analysis was performed using STATA/IC version 13.1 (StataCorp, Texas). Descriptive statistical analysis was depicted as median and inter-quantile range or means and standard deviation as appropriate. Body mass index (kg/m2) was classified as underweight (BMI <18), normal weight (BMI 18–24.9), overweight (BMI 25–34.9) and obesity (BMI> 35). The per-protocol population was used for primary efficacy analysis including all participants who completed one of the respective treatment groups (including one patient who inadvertedly switched treatment group) and who were followed-up until the end of the study, (Fig 1). The proportions of participants with *L*. *loa* microfilaraemia ≤ 100mf/ml were presented by study group using a frequency table with a 95% confidence interval. The albendazole group was chosen as reference to compute odds ratios for achieving the primary endpoint. The reduction in microfilaraemia per group was calculated as the median of the relative reduction in microfilaraemia per each participant. Area under the curve of log-10 transformed *L*. *loa* microfilaraemia from baseline until D168 by the trapezoidal rule has been calculated with 95% confidence intervals. The number and percentage of participants who experienced adverse events in the safety population consisting of all patients receiving study treatment were tabulated by study group.

## Results

### Study population and protocol adherence

From May 2018 to April 2020 a total of 78 patients positive for *L*. *loa* microfilariae were screened for eligibility into the study. 49 eligible patients were randomized into the different study groups of whom three dropped out prior to treatment initiation because of withdrawal of consent and 4 patients in the ALB+ALB group discontinued treatment (2 patients moved out of the study area and 2 participants refused to continue the study due to repeated blood sampling during the treatment period). These 7 individuals were replaced by the next available eligible patient. Overall, 36 randomized patients (12 in each of the intervention groups) completed their first 21 days of ALB treatment. Two patients randomized to the ALB+IVM group received follow-up treatment with an additional 14 days of albendazole, instead of a dose of ivermectin, because their microfilaraemia was still above the protocol-specified threshold of 4,000/ml (*L*. *loa* microfilaraemia were at 41,500 mf/ml and 7,450 mf/ml respectively after the first 21 days of albendazole treatment), as specified by the study protocol. These patients were allocated to the ALB+ALB group for statistical analysis. Moreover, one patient randomized in the ALB+ALB group was inadvertedly treated with ivermectin after the initial 21 days of albendazole and was therefore pragmatically considered for statistical analysis in the ALB+IVM group. Six patients in the control group received loratadine for 7 days. The 40 patients who received at least one dose of albendazole, plus the 6 patients in the control group, together constitute the safety population for the safety analysis. One in the ALB group was lost to follow-up after Day 154 because of migration out of the study region and two patients in the ALB+IVM group were lost to follow-up after visits on Day 37 and Day 154 due to withdrawal of consent and migration out of the study region. All other patients were followed-up for 168 days. The per-protocol population for the primary efficacy analysis therefore consists of the 39 patients who completed one of the treatment groups as well as the full 168-day follow-up period. Details of participants’ flow are depicted in the Fig 1.

### Baseline characteristics

As shown in Table 1, the study population had a median age of 63 years (IQR: 51–72), the gender was well balanced with as many women as men overall. The majority of patients, 67% (28/42), were within the normal range of BMI and 17% (7/42) were overweight. Median blood pressure was borderline or elevated in all treatment groups. The median load of microfilariae was similar between the groups at baseline, as were the haemoglobin levels and the eosinophil counts (Table 1).

**Table 1:** Baseline demographic, clinical and biological characteristics of the safety population (N = 46).

		ALB	ALB+ALB	ALB+IVM	Control	Total
**N**		12	16	12	6	46
**Age (years), median (IQR)**		68 (62–78)	60 (47–71)	62 (57–72)	52 (47–63)	63 (51–72)
**Sex (Female/Male)**		7/5	8/8	5/7	2/4	22/24
**Weight (kg), median (IQR)**		61.5 (50.0–75.0) ***	60.0 (49.5–70.0)	61.5 (49.5–70.0)	58.0 (55.0–66.0)	60.5 (50.0–70.0)***
**BMI (kg/m** ^**2**^ **), median (IQR)**		24.8 (22.2–28.4) ***	22.0 (20.1–25.7) *	23.3 (20.2–24.3)	21.5 (19.0–22.8)	22.5 (20.2–25.7)****
**BMI by category**						
	Underweight, n (%)	0	2 (13.3)	1 (8.3)	0	3 (7.1)
	Normal, n (%)	5 (55.6)	9 (60.0)	9 (75.0)	5 (83.3)	28 (66.7)
	Overweight, n (%)	3 (33.3)	2 (13.3)	2 (16.7)	0	7 (16.7)
	Obese, n (%)	1 (11.1)	2 (13.3)	0	1 (16.7)	4 (9.5)
**Temperature (°C), mean (sd)**		36.5 (0.3)	36.5 (0.7)	36.2 (0.4)	36.5 (0.3)	36.4 (51–72)
**Blood pressure(mmHg)**						
	SBP, mean (sd)	149.2 (24.9)	143.1 (24.8)	155.0 (28.8)	136.7 (13.5)	146.9 (24.8)
	DBP, mean (sd)	85.3 (15.5)	87.6 (15.0)	95.1 (14.0)	78 (5.7)	87.7 (14.6)
Median Baseline LLM (parasite/ml), (IQR)		9625 (8050–12850)	9225 (7300–16025)	12725 (6600–17550)	11900 (7500–17350)	9950 (7500–15550)
Laboratory parameters, median (IQR)						
	RBC count, (10^6^/mm^3^)	4.9 (4.1–5.6)	4.6 (4.2–5.1)	4.7 (3.9–5.4)	4.3 (4.0–4.8)	4.6 (4.1–5.1)
	Hb, (g/dl)	12.8 (11.4–13.5)	12.9 (11.1–13.4)	12.6 (11.3–14.5)	13.0 (11.9–14.2)	12.9 (11.4–13.6)
	WBC count, (10^3^/mm^3^)	6.7 (5.9–10.5)	6.2 (5.7–9.2)	8.0 (6.6–9.4)	7.7 (6.2–10.9)	7.1 (5.9–9.2)
	Platelets, (10^3^/mm^3^)	189 (169–281)	208 (113–267)	199 (132–252)	222 (191–243)	204 (154–259)
	Eosinophils, (percentage of total WBC in differential blood count)	15.9 (12.4–20.3)	14.6 (11.8–16.9)	16.5 (13.5–22.2)	16.7 (12.2–17.6)	15.7 (12.4–20.2)
	ASAT, (mmol/ml)	21.4 (18.8–25.5)	23.6 (19.2–30.6)	23.2 (18.7–30.0)	24.8 (21.6–28)	23.0 (19.3–28.0)
	ALAT, (mmol/ml)	14.5 (11.3–15.5)	14.4 (8.5–19.8)	13.0 (11.2–15.0)	14.6 (10–16.3)	14.0 (10.5–13.5)

Abbreviations: ALB: albendazole; IVM: ivermectin; BMI: body mass index; SBP: systolic blood pressure, DBP: diastolic blood pressure, LLM: *Loa loa* microfilaraemia; sd: standard deviation; RBC: Red Blood Cell, Hb: haemoglobin, WBC: white blood cell, ASAT: aspartate aminotransferase, ALAT: alanine aminotransferase, ****: 4 missing data, ***: 3 missing data; *: 1 missing data

### Proportion of participants with *L*. *loa* microfilaraemia ≤100mf/mL at 6-month follow-up (Day 168)

A total of 39 participants were included in this analysis contributing data until the end of follow-up on Day 168. The highest proportion of individuals with blood microfilariae loads below 100/ml were seen in the ALB+ALB group (39%, 5/13) followed by ALB+IVM group (22%, 2/9). None of the patients in the control group had a microfilarial load below 100mf/ml (Table 2).

**Table 2:** Proportion of participants with *L*. *loa* ≤ 100mf/ml at 6-month.

	n/N	Proportion (95%CI)	OR (95%CI)
**ALB**	1/11	9 (0–26)	1
**ALB+ALB**	5/13	39 (12–65)	6 (1–65)
**ALB+IVM**	2/9	22 (0–49)	3 (0–38)
**control**	0/6	0	--

Abbreviations: ALB: albendazole; IVM: ivermectin; OR: odd ratio; CI: confident interval; n/N: ration of number of subject with event by number of subject in group

Four patients had no detectable microfilariae in the blood at 6-month follow-up, 1 out of 11 (9%) in the ALB group and 3 out 13 (23%) in the ALB+ALB group.

### Time to nadir and evolution of median microfilarial load

**Fig 2:**
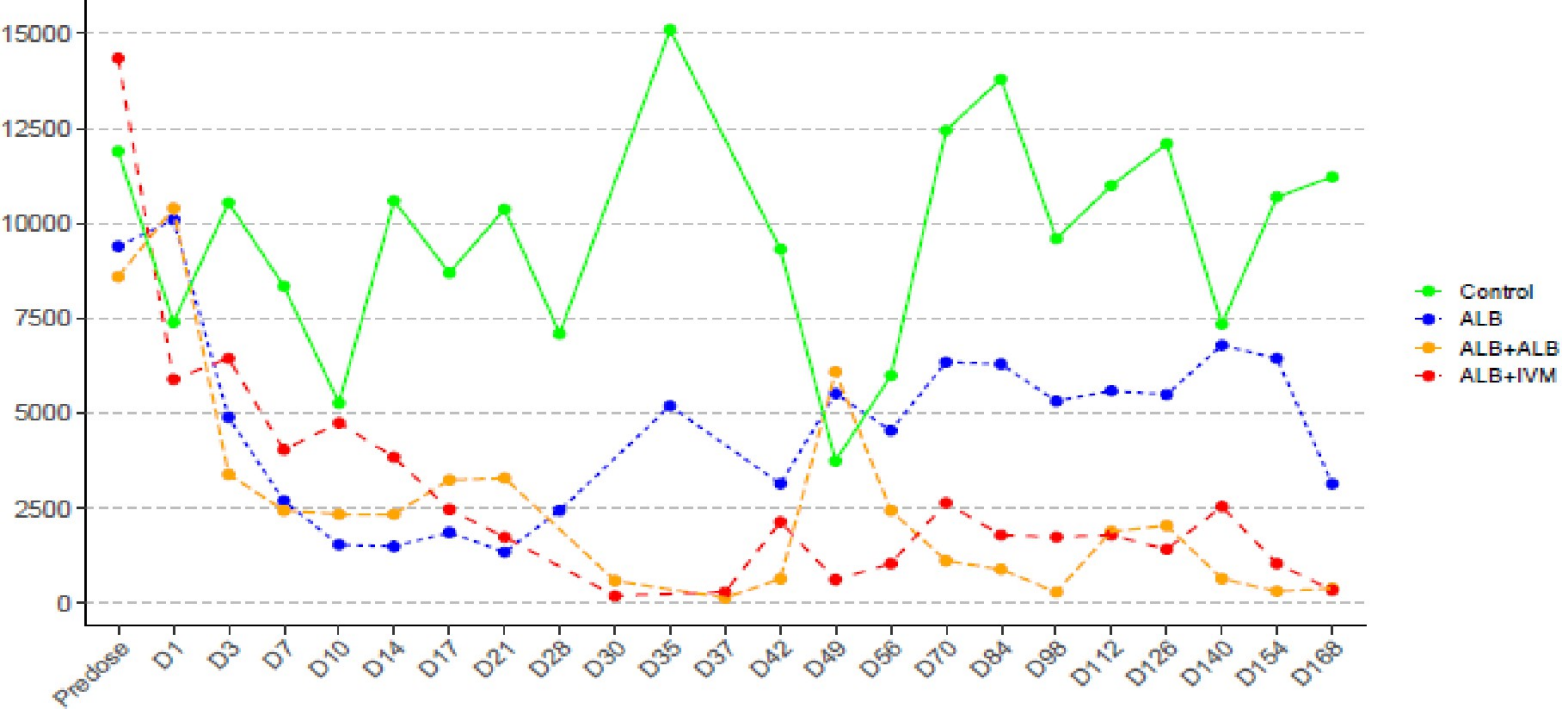
Loa loa median microfilaraemia load variation from baseline to the end of follow-up according to study group. Abbreviations: ALB: albendazole; IVM: ivermectin

While the median microfilarial load for all treatment groups at baseline was around 10,000 per ml as shown in Table 1, the median of the lowest microfilarial load was reached at Day 21, Day 30 and Day 37 following initiation of treatment in the intervention groups ALB group, ALB+IVM group and ALB+ALB group, respectively (Fig 2). For the remaining follow-up period following individuals’ nadir, the median microfilarial load remained constantly below 2,500 per ml for the treatment groups ALB+IVM and ALB+ALB while it went up to around 5,000 per 100/ml for the ALB group. The control group showed a fluctuation in microfilarial load that remained above 5,000 per ml throughout the study period. Details of the dynamics of microfilarial load from baseline for all study groups are shown in Fig 2.

### Median reduction in *L*. *loa* microfilariae at each month from baseline

To compare the activity of each treatment regimen on microfilarial load for each respective group, the relative reduction in median microfilarial load (IQR) was computed. The relative reduction from baseline was >90% in the ALB+ALB and the ALB+IVM groups starting from around one-month post-treatment initiation with some small fluctuations during the 6-month follow-up. The ALB group showed a slightly lower relative reduction of about 80% from one-month post-treatment—also observed at the end of the 6-month follow-up period—while there were also considerable inter-individual variations throughout the follow-up period. The control group did not show any clinically significant relative reduction in median microfilarial load throughout the follow-up period. Details are shown in Table 3.

**Table 3:** Median (IQR) of relative reduction in *L*. *loa* mf per month by study group.

	ALB	ALB+ALB	ALB+IVM	Control
**M1**	0.80 (0.59–0.88)	0.91 (0.44–0.99)	0.96 (0.93–0.99)	0.23 (0.15–0.61)
**M2**	0.60 (0.12–0.82)	0.71 (-0.42–0.71)	0.87 (0.69–0.99)	0.13 (-0.01–0.20)
**M3**	0.47 (0.03–0.77)	0.96 (0.58–0.99)	0.84 (0.47–1)	0.10 (0.05–0.10)
**M4**	0.47 (0.46–0.92)	0.88 (0.62–0.97)	0.90 (0.70–0.98)	-0.16 (-0.06–0.54)
**M5**	0.27 (-0.20–0.85)	0.98 (-0.12–0.99)	0.86 (0.68–0.95)	0.15 (0.06–0.72)
**M6**	0.69 (0.57–0.85)	0.93 (0.87–0.99)	0.94 (0.85–0.97)	0.02 (-0.38–0.35)

Abbreviations: ALB: albendazole; IVM: ivermectin; M: month

Area under the curve (AUC) of microfilaraemia was calculated from baseline until D168 in the respective treatment groups as in Table 4. The lowest AUC were seen in the ALB+ALB group and the ALB+IVM group with 448 (327 to 570) and 498 (386 to 611), respectively.

**Table 4:** Area under the curve of microfilaraemia in the respective treatment groups over 6 months follow-up.

Treatment groups	n	AUC (Mean; (95% CI))
**Control**	6	663 (623–703)
**ALB**	11	566 (504–627)
**ALB+ALB**	13	448 (327–570)
**ALB+IVM**	9	498 (386–611)

Abbreviations: ALB: albendazole; IVM: ivermectin; n, number, AUC: area under the cuve, CI: confident interval

### Safety analysis

Of the 46 patients included in the safety population that either received one or more doses of albendazole (40 patients) or were enrolled in the control group (6 patients) a total of 15 (32.6%) reported one or more adverse events at least possibly related to study drugs, including just one patient in the control group, six in the ALB group and four each in the ALB+ALB and the ALB+IVM groups. The most commonly reported at least possibly study drug related adverse events occurred within the first 3 weeks of treatment and were headache, dizziness and fatigue, followed by pruritus, rash and nausea as shown in Table 5. Out of 4 patients randomized to the ALB+ALB group who were lost during treatment period, only one of them reported an adverse event before leaving the study, which was headache, grade I and unlikely related to study drug.

**Table 5:** Grade 1–2 adverse events at least possibly related to study treatment.

Adverse event, n (%)	ALB (N = 12)	ALB+ALB (N = 16)	ALB+IVM (N = 12)	Control (N = 6)	Total (N = 46)
**Patient with any at least possibly related AE**	6 (50.0)	4 (25.0)	4 (33.3)	1 (16.6)	15 (32.6)
**Number of AEs at least possibly related to study drug**	8	7	6	1	22
**Headache**	0	4 (57.1)	0	0	4 (18.2)
**Dizziness**	1 (12.5)	1 (14.3)	2 (33.3)	0	4 (18.2)
**Vertigo**	0	1 (14.3)	0	0	1 (4.6)
**Nausea**	0	0	2 (33.3)	0	2 (9.1)
**Abdominal pain**	0	0	0	1 (100)	1 (4.6)
**Diarrhoea**	1 (12.5)				1 (4.6)
**Fatigue**	3 (37.5)	0	1 (16.7)	0	4 (18.2)
**Pruritus**	1 (12.5)	1 (14.3)	1 (16.7)		3 (13.6)
**Rash**	2 (25.0)	0	0	0	2 (9.1)

Abbreviations: ALB: albendazole; IVM: ivermectin; AEs: adverse event

Among all patients in the safety analysis there were no AEs rated at grade 3 or higher except for one patient with a serious adverse event. This 73-years-old man with a history of alcohol abuse was randomized to the ALB group with an initial *L*. *loa* micofilaraemia of 10,050 mf/ml. Baseline haematology and biochemistry parameters were normal, except for gamma glutamine transferase at 1.5 time upper normal limit. One day before the end of the three weeks albendazole drug intake, the patient became asthenic with psychomotor retardation without fever. Five days later the decision was taken to hospitalize the patient due to a new onset of vertigo and aggravation of asthenia. At hospital admission, the patient was afebrile, Glasgow 5/5 and asthenic. Neurological examination was normal. Haematology and biochemistry assessments were unremarkable, except for C-reactive protein and gamma glutamine transferase which were 3 and 2 times upper normal limit, respectively. *L*. *loa* microfilaria count was 1,400mf/ml. Symptomatic supportive treatment was given and the patient was discharged four days later in full remission with the suspicion of alcohol withdrawal syndrome as the underlying cause of the symptoms. This serious adverse event was classified as unlikely related to study treatment.

The evolution of haematological parameters showed a consistent trend in all active treatment groups with a slight decrease of haemoglobin values from baseline to Day-21 which resolved by day 168. At the same time white blood cell counts increased during the active treatment period before reaching initial levels at the end of follow-up. Importantly, relative eosinophil count increased in all treatment groups before decreasing well below the initial value at the end of the observation period. All these changes were similar in the active treatment groups and were not observed in the CONTROL group. (Table 6)

**Table 6:** Haematology and biochemistry parameters before and after study drug administration in each group.

Median (IQR)		ALB			ALB+ALB			ALB+IVM			Control		
		SCR	D21	D168	SCR	D21	D168	SCR	D21	D168	SCR	D21	D168
Haematological parameters (IQR)													
	RBC, **(**10^6^/mm^3^)	4.9 (4.1–5.6)	4.2 (3.9–4.8)	4.4 (4.1–4.7)	4.6 (4.2–5.1)	3.9 (3.8–4.8)	4.8 (4.2–5.1)	4.7 (3.9–5.4)	4.3 (4.0–4.5)	4.7 (4.2–5.1)	4.3 (4.0–4.8)	4.6 (4.2–4.8)	4.6 (4.5–4.6)
	Hb, (g/dl)	12.8 (11.4–13.5)	11.5 (11.0–12.5)	11.6 (11.3–12.7)	12.9 (11.1–13.4)	11.7 (9.7–12.1)	11.9 (11.0–13.6)	12.6 (11.3–14.5)	12.4 (10.9–13.4)	12.3 (11.3–13.2)	13.0 (11.9–14.2)	13.2 (11.8–13.5)	13.9 (13.5–14.4)
	WBC, (10^3^/mm^3^)	6.7 (5.9–10.5)	9.0 (7.7–10.9)	7.3 (5.1–7.9)	6.2 (5.7–9.2)	8.7 (6.3–18.1)	5.3 (4.8–9.9)	8.0 (6.6–9.4)	12.0 (10.3–12.8)	6.3 (5.0–7.3)	7.7 (6.2–10.9)	9.1 (7.8–10.4)	9.1 (7.8–10.4)
	Platelets^c^, (10^3^/mm^3^)	189 (169–281)	191 (87–278)	180 (103–245)	208 (113–267)	231 (138–309)	108 (70–168)	199 (132–252)	210 (109–226)	213 (148–274)	221 (191–243)	232 (212–271)	232 (212–271)
	EOSI,(% of total WBC in differential blood count)	15.9 (12.4–20.3)	18.2 (9.0–32.8)	5.5 (4.9–6.0)	14.6 (11.8–16.9)	23.3 (20.9–30.0)	5.3 (4.8–6.6)	16.5 (13.5–22.2)	23.3 (15.8–29.8)	5.1 (3.0–7.9)	16.7 (12.2–17.6)	22.3 (13.6–24.8)	13.3 (7.5–22.6)
Biochemistry parameters (IQR)													
	ASAT, (mmol/l)	21.4 (18.8–25.5)	32.0 (23.0–40.4)	20.1 (18.0–22.8)	23.6 (19.2–30.6)	48.9 (31.5–66.8)	35.6 (27.6–48.2)	23.2 (18.7–30.0)	43.6 (23.1–56.5)	28.0 (19.5–39.6)	24.8 (21.6–28)	23.9 (19.4–27.5)	21.3 (23.1–56.5)
	ALAT, (mmol/l)	14.5 (11.3–15.5)	29.0 (19.6–45.0)	11.5 (8.9–15.7)	14.4 (8.5–19.8)	29.3 (16.6–54.9)	17.7 (14.9–26.9)	13.0 (11.2–15.0)	18.0 (34.3–50.6)	12.6 (9.9–17.6)	14.6 (10–16.3)	15.0 (14.4–15.1)	13.6 (12.7–17.3)

Abbreviations: ALB: albendazole; IVM: ivermectin; RBC: red blood cell, Hb: haemoglobin, WBC: white blood cell, EOSI: Eosinophils, ASAT: aspartate aminotransferase, ALAT: alanine aminotransferase, SCR: Screening, D: day

A slight increase in liver transaminases (ALT and AST) was observed following 21 days of albendazole intake in all treatment groups, but not in the control group. Nevertheless, no further increase was observed after an additional course of 14 days of albendazole in the ALB+ALB group, nor after administration of a single dose of ivermectin in ALB+IVM. (Table 6).

## Discussion

Safe and effective treatment regimens that can be used without specific biological monitoring of patients are necessary for the development of drug-based control programs for loiasis. Currently albendazole based drug regimens that are known to be relatively safe in gradually reducing microfilarial load are the only viable options due to safety concerns for the use of DEC and ivermectin when used without detailed biological screening and close follow-up in patients with loiasis [23–25].

This clinical trial systematically assessed the parasitological outcomes of three different albendazole-based regimens on *L*. *loa* microfilaraemia when compared to an inactive control group. Albendazole given for 5 weeks and albendazole for three weeks followed by a single dose ivermectin treatment appeared to be the most efficacious treatment regimens with 39% and 22% reaching the primary outcome measure of microfilaraemia below 100mf/ml at 6-months. Concordantly, the AUC of microfilaraemia indicated that the ALB+ALB and the ALB+IVM regimens led to the most consistent reduction of microfilaraemia over time. Albendazole is thought not to exert a significant direct activity on *L*. *loa* microfilariae, but rather on the female adult worms by inhibiting the reproductive process and direct adulticidal activity in prolonged regimens. Interestingly, the three-week albendazole treatment regimen alone led to a similar decrease during the active treatment period followed by a subsequent rebound in microfilaraemia. This loss of suppressive action in the ALB group was not reported in other retrospective cohorts in Gabon but may be explained by the above-mentioned mode of action of albendazole by insufficient or transient sterilisation of female worms, potential reinfections during the study period, incomplete adherence to the drug regimen, or the high level of transmission of loiasis in the study region. The addition of a single dose of ivermectin has a similar effect as prolongation of albendazole therapy alone for another 2 weeks and was similarly not associated with complete suppression of microfilaraemia. Importantly, adding a single dose of invermectin constitutes a logistical advantage compared to a further two weeks of albendazole intake in terms of treatment adherence and treatment costs. Contrarily to this finding a cohort of patients with imported loiasis in Italy, where reinfection of individuals was no longer possible, showed complete cure with an albendazole-ivermectin based regimen [25]. Whereas the reason for this discrepancy are currently only incompletely understood, one reason may lie in higher microfilarial load, the potentially higher number of adult worms and a younger adult worm population due to the ongoing transmission in our study participants. It may here only be speculated about the adulticidal effect of the treatment regimens as no clear markers for clearance of adult *L*. *loa* filariae are available. Whereas albendazole is thought to act first on the fecundity of female adult worms, an adulticidal effect has been postulated for longer and high dose regimens. Contrarily, ivermectin is thought to primarily act by supressing the microfilarial load without importantly affecting the adult worms. [11–13, 21, 22]

Overall all treatment regimens proved safe and well tolerated in this relatively small clinical trial. One SAE was retrospectively judged to be associated with an undisclosed alcohol withdrawal syndrome and no other significant safety findings where observed. Various manifestations have been attributed to loiasis including and a probable case of encephalopathy due to loiasis in the absence of any treatment [6, 26, 27]. However, we cannot definitively rule out the possibility that this SAE could be caused by treatment with albendazole even if the event developed three weeks after initiation of treatment.

The transient increase in liver transaminases and increases in relative eosinophil counts paralleled by a modest reduction of haemoglobin during the active treatment period are well-described reversible side effects of high dose albendazole therapy [14, 23, 24, 28, 29]. No clinically important or treatment limiting adverse drug reaction was observed. However, the small sample size limits the generalizability of this reassuring safety finding as evidenced by previous reports of encephalopathy following albendazole therapy in patients suffering from loiasis [25–27]. The inclusion of relatively healthy individuals in this clinical trial also constitutes a limitation for the external validity of this study in the general population. Importantly, liver enzymes and haemoglobin levels normalized at the end of the follow-up period and markedly decreased eosinophil counts are indirect markers for the effective cure of loiasis and potentially other concomitant helminthic infections. Since a proportion of patients intolerable to long-term albendazole therapy with clinically significant liver toxicity or transient hematological side effects has been reported, repeated monitoring of biochemistry and hematology is necessary to safely use this drug.

It is remarkable that the study population was rather old with a median age above 60 years. This finding is at least partly explained by the fact that older individuals are overrepresented in rural Gabonese villages compared to the national average and that the prevalence of loiasis and to a certain extent the level of microfilaraemia increases with age in our setting. In addition, the professional activities of rural populations may have discouraged participation in this clinical trial due to a demanding study schedule and a prolonged follow up period. The main limitation of this study is its descriptive study design with the limited number of participants included precluding formal statistical comparison between treatment groups in this proof of concept trial. Whereas this study systematically describes the effect of the treatment regimens over a 6-month period, no further conclusions may be drawn for a longer duration of the effect beyond the 6-month follow-up period. Similarly, only a fraction of participants has reached the pre-specified threshold of 100mf/ml, which was set as measure to reduce onwards transmission of loiasis. Further epidemiological and experimental data on the infectivity of loiasis at respective microfilarial loads are required to corroborate robust endpoints for clinical trials aiming at reducing loiasis transmission in highly endemic regions [30]. The assessment of *L*. *loa* microfilaraemia from both venous and peripheral blood samples is another limitation of this study. However, based on the natural diurnal variation of microfilarial load and the consistent blood drawing schedule between treatment groups, no systematic confounding was introduced between treatment regimens. Finally, the evaluation of treatment response from a patient’s perspective should be performed in future evaluations of individual treatment outcomes.

## Conclusions

In summary, the efficacy of albendazole based regimens in clearing microfilaraemia seems to be limited to an approximate 90% reduction in this high transmission area. Its slow onset of action over several weeks makes it suitable for the safe and gradual reduction of microfilaraemia. However, at the same time the requirement for prolonged treatment regimens over 3–5 weeks is an important limitation for its large-scale use in population-based treatment programmes. Alternative drugs or drug formulations are therefore needed to simplify treatment regimens and to provide the tools for community-based control programmes in *L*. *loa* endemic area. Given that clinical trials on loiasis treatment are scare, adding systematic information on the safety and efficacy of existing drugs in an endemic area is useful and may help standardizing treatment regimens for the control of *L*. *loa* transmission.

## Supporting information

S1 Consort Checklist CONSORT 2010 checklist of information to include when reporting a randomised trial. (DOC)

S1 Data Underlying data. (XLSX)

S1 Study Protocol Safety and efficacy of different albendazole-based treatment regimens to reduce microfilaraemia in subjects infected by *Loa loa* in an endemic area of Gabon: A randomised controlled open-label pilot study. (DOCX)

## References

[1] Loiasis Boussinesq M. Ann Trop Med Parasitol. Dec 2006;100(8):715–31. 17227650 10.1179/136485906X112194

[2] Metzger WG, Mordmüller B. Loa loa-does it deserve to be neglected? Lancet Infect Dis. Avr 2014;14(4):353–7. doi: 10.1016/S1473-3099(13)70263-9 24332895

[3] Zouré HGM, Wanji S, Noma M, Amazigo UV, Diggle PJ, Tekle AH, et al. The geographic distribution of Loa loa in Africa: results of large-scale implementation of the Rapid Assessment Procedure for Loiasis (RAPLOA). PLoS Negl Trop Dis. Jun 2011;5(6):e1210. 21738809 10.1371/journal.pntd.0001210 PMC3125145

[4] Prevention CC for DC and. CDC—Loiasis—Resources for Health Professionals [Internet]. 2020 [cited 6 Oct 2021]. Available on: https://www.cdc.gov/parasites/loiasis/health_professionals/index.html.

[5] Loiasis (African Eye Worm) Treatment & Management: Medical Care, Surgical Care, Complications. 20 July 2021 [cited 5 Oct 2021]; Available on: https://emedicine.medscape.com/article/2500105-treatment.

[6] Veletzky L, Hergeth J, Stelzl DR, Mischlinger J, Manego RZ, Mombo-Ngoma G, et al. Burden of disease in Gabon caused by loiasis: a cross-sectional survey. Lancet Infect Dis. 1 Nov 2020;20(11):1339–46. doi: 10.1016/S1473-3099(20)30256-5 32585133

[7] Chesnais CB, Pion SD, Boullé C, Gardon J, Gardon-Wendel N, Fokom-Domgue J, et al. Individual risk of post-ivermectin serious adverse events in subjects infected with Loa loa. EClinicalMedicine [Internet]. 1 Nov 2020 [cited 6 oct 2021];28. Available on: https://www.thelancet.com/journals/eclinm/article/PIIS2589-5370(20)30326-6/fulltext. doi: 10.1016/j.eclinm.2020.100582 33294807 PMC7700892

[8] Anderson J, Fuglsang H, de C Marshall TF. Effects of diethylcarbamazine on ocular onchocerciasis. Tropenmed Parasitol. Sept 1976;27(3):263–78. 982546

[9] Bryceson AD, Warrell DA, Pope HM. Dangerous reactions to treatment of onchocerciasis with diethylcarbamazine. Br Med J. 19 March 1977;1(6063):742–4. doi: 10.1136/bmj.1.6063.742 851711 PMC1605656

[10] Crump A, Ōmura S. Ivermectin, ‘Wonder drug’ from Japan: the human use perspective. Proc Jpn Acad Ser B Phys Biol Sci. 10 Feb 2011;87(2):13. doi: 10.2183/pjab.87.13 21321478 PMC3043740

[11] Boussinesq M. Loiasis: new epidemiologic insights and proposed treatment strategy. J Travel Med. Jun 2012;19(3):140–3. doi: 10.1111/j.1708-8305.2012.00605.x 22530819

[12] Kamgno J, Boussinesq M. Effect of a single dose (600 mg) of albendazole on Loa loa microfilaraemia. Parasite Paris Fr. March 2002;9(1):59–63. doi: 10.1051/parasite/200209159 11938697

[13] Kamgno J, Nguipdop-Djomo P, Gounoue R, Téjiokem M, Kuesel AC. Effect of Two or Six Doses 800 mg of Albendazole Every Two Months on Loa loa Microfilaraemia: A Double Blind, Randomized, Placebo-Controlled Trial. PLoS Negl Trop Dis [Internet]. 11 mars 2016 [cited 22 Feb 2020];10(3). Available on: https://www.ncbi.nlm.nih.gov/pmc/articles/PMC4788450/. 10.1371/journal.pntd.0004492 PMC4788450 26967331

[14] Tabi TE, Befidi-Mengue R, Nutman TB, Horton J, Folefack A, Pensia E, et al. Human loiasis in a Cameroonian village: a double-blind, placebo-controlled, crossover clinical trial of a three-day albendazole regimen. Am J Trop Med Hyg. August 2004;71(2):211–5. 15306713

[15] Tsague-Dongmo L, Kamgno J, Pion SDS, Moyou-Somo R, Boussinesq M. Effects of a 3-day regimen of albendazole (800 mg daily) on Loa loa microfilaraemia. Ann Trop Med Parasitol. Oct 2002;96(7):707–15. doi: 10.1179/000349802125001933 12537632

[16] Manego RZ, Mombo-Ngoma G, Witte M, Held J, Gmeiner M, Gebru T, et al. Demography, maternal health and the epidemiology of malaria and other major infectious diseases in the rural department Tsamba-Magotsi, Ngounie Province, in central African Gabon. BMC Public Health. 28 2017;17(1):130. doi: 10.1186/s12889-017-4045-x 28129759 PMC5273856

[17] Ramharter M, Agnandji ST, Adegnika AA, Lell B, Mombo-Ngoma G, Grobusch MP, et al. Development of sustainable research excellence with a global perspective on infectious diseases: Centre de Recherches Médicales de Lambaréné (CERMEL), Gabon. Wien Klin Wochenschr. 2021;133(9):500. 33398458 10.1007/s00508-020-01794-8 PMC7781170

[18] Joanny F, Löhr SJZ, Engleitner T, Lell B, Mordmüller B. Limit of blank and limit of detection of Plasmodium falciparum thick blood smear microscopy in a routine setting in Central Africa. Malar J. 14 Jun 2014;13:234. doi: 10.1186/1475-2875-13-234 24929248 PMC4069274

[19] Mischlinger J, Manego RZ, Mombo-Ngoma G, Mbassi DE, Hackbarth N, Mbassi FAE, et al. Diagnostic performance of capillary and venous blood samples in the detection of Loa loa and Mansonella perstans microfilaraemia using light microscopy. PLoS Negl Trop Dis. August 2021;15(8):e0009623. doi: 10.1371/journal.pntd.0009623 34398886 PMC8389422

[20] Sandri TL, Kreidenweiss A, Cavallo S, Weber D, Juhas S, Rodi M, et al. Molecular Epidemiology of Mansonella Species in Gabon. J Infect Dis. 3 Feb 2021;223(2):287–96. doi: 10.1093/infdis/jiaa670 33099649

[21] Kershaw WE, Chalmers TA, Duke BOL. Studies on the Intake of Microfilariae by their Insect Vectors, their Survival, and their Effect on the Survival of their Vectors. Ann Trop Med Parasitol. 1 Sept 1954;48(3):329–39. 13208162 10.1080/00034983.1954.11685631

[22] Harris PA, Taylor R, Thielke R, Payne J, Gonzalez N, Conde JG. Research electronic data capture (REDCap)—a metadata-driven methodology and workflow process for providing translational research informatics support. J Biomed Inform. Apr 2009;42(2):377–81. doi: 10.1016/j.jbi.2008.08.010 18929686 PMC2700030

[23] Klion AD, Massougbodji A, Horton J, Ekoué S, Lanmasso T, Ahouissou NL, et al. Albendazole in human loiasis: results of a double-blind, placebo-controlled trial. J Infect Dis. July 1993;168(1):202–6. doi: 10.1093/infdis/168.1.202 8515109

[24] Gobbi F, Buonfrate D, Tamarozzi F, Degani M, Angheben A, Bisoffi Z. Efficacy of High-Dose Albendazole with Ivermectin for Treating Imported Loiasis, Italy. Emerg Infect Dis. August 2019;25(8):1574–6. doi: 10.3201/eid2508.190011 31310225 PMC6649345

[25] Gobbi F, Bottieau E, Bouchaud O, Buonfrate D, Salvador F, Rojo-Marcos G, et al. Comparison of different drug regimens for the treatment of loiasis—A TropNet retrospective study. PLoS Negl Trop Dis [Internet]. 1 nov 2018 [cited 22 Feb 2020];12(11). Available on: https://www.ncbi.nlm.nih.gov/pmc/articles/PMC6233929/. doi: 10.1371/journal.pntd.0006917 30383753 PMC6233929

[26] Arrey-Agbor DB, Nana-Djeunga HC, Mogoung-Wafo AE, Mafo M, Danwe C, Kamgno J. Case Report: Probable Case of Spontaneous Encephalopathy Due to Loiasis and Dramatic Reduction of Loa loa Microfilariaemia with Prolonged Repeated Courses of Albendazole. Am J Trop Med Hyg. July 2018;99(1):112–5. doi: 10.4269/ajtmh.17-0664 29741149 PMC6085801

[27] Lukiana T, Mandina M, Situakibanza NH, Mbula MM, Lepira BF, Odio WT, et al. A possible case of spontaneous Loa loa encephalopathy associated with a glomerulopathy. Filaria J. 10 May 2006;5:6. doi: 10.1186/1475-2883-5-6 16686951 PMC1471781

[28] Klion AD, Horton J, Nutman TB. Albendazole therapy for loiasis refractory to diethylcarbamazine treatment. Clin Infect Dis Off Publ Infect Dis Soc Am. Sept 1999;29(3):680–2. doi: 10.1086/598654 10530467

[29] Baraka V, Ishengoma DS, Fransis F, Minja DTR, Madebe RA, Ngatunga D, et al. High-level Plasmodium falciparum sulfadoxine-pyrimethamine resistance with the concomitant occurrence of septuple haplotype in Tanzania. Malar J. 2015;14:439. doi: 10.1186/s12936-015-0977-8 26542942 PMC4635599

[30] Whittaker C, Walker M, Pion SDS, Chesnais CB, Boussinesq M, Basáñez MG. The Population Biology and Transmission Dynamics of Loa loa. Trends Parasitol. 1 Apr 2018;34(4):335–50. doi: 10.1016/j.pt.2017.12.003 29331268

