# Supplementary material for: Efficacy, safety, and tolerability of albendazole and ivermectin based regimens for the treatment of microfilaraemic loiasis in adult patients in Gabon: A randomized controlled assessor blinded clinical trial
Source: PLoS Negl Trop Dis. 2023 Aug 28;17(8):e0011584. doi: 10.1371/journal.pntd.0011584 (PMC10491396; doi:10.1371/journal.pntd.0011584)
Supplement: S1 Study Protocol — (DOCX) [file pntd.0011584.s003.docx]

**Clinical Trial Protocol**

Safety and efficacy of different albendazole-based treatment regimens to reduce microfilaraemia in subjects infected by *Loa loa* in an endemic area of Gabon:

a randomised controlled open-label pilot study

Protocol version 3 (08-06-2018)

Sponsor: Prof. Ayôla Akim Adegnika, MD PhD

Principal investigator: Prof. Michael Ramharter, MD

Local Principal Investigator: Dr. Rella Zoleko Manego, MD

Co-investigators: Dr. Matthew McCall, MD PhD DTMH

Dr. Ghyslain Mombo-Ngoma, MD PhD

Dr. Luzia Veletzky, MD

Dr. Wolfram Metzger, MD

Ass Prof Dr. Heimo Lagler, MD, MPH

Prof. Benjamin Mordmüller, MD

Prof. Peter G Kremsner, MD PhD

Centre de Recherches Médicales de Lambaréné (CERMEL)

B.P. 242, Lambaréné

Gabon

# Document history

| **Version (date)** | **Changes** | **Author** |
| --- | --- | --- |
| V1.0 (06-09-2017) | Original version | Rella Zoleko Manego  Matthew McCall |
| V1.1 (08-09-2017) | Update of the trial design and other modifications of the protocol, English + French summary added | Michael Ramharter  Matthew McCall |
| V1.2 (11-10-2017) | Modifications based on CEI review: addition of voluntary treatment option for arm 1 at end of study; addition of recent albendazole intake as exclusion criterion; clarification of (frequency of) follow-up visits in arms 3+4; clarification/addition of blood sampling time points (during initial ‘treatment’ phase in arm 1, at DRT+1 in arm 4 and at month 6 in all study arms); clarification of blood sample volumes; clarification of symptomatic treatment; addition of PCR as mf detection/quantification method; addition of paragraph on subject risk/benefit; limitation of sample storage duration to 5 years; clarification of study monitoring. |  |
| V2(22-01-2018) | Modification of threshold of parasitemia as inclusion criteria: For reasons of patient safety, we have set an upper limit of 50.000mf/l, and the benefit of treatment is also expected for patients with parasitemia less than 10,000 mf / μl. Moreover, for logistical reasons (too far/isolated), refusal to participate we may reduce the threshold of parasitemia below 10.000 mf/µl  Addition of stool and urine samples collection before treatment: We wish to have the intestinal parasitic status of the participants since we will treat with albendazole which also has an effect on many helminths; which could affect the tolerability and influence nonspecific symptoms such as pruritus. Moreover for the immunological evaluation the search of these parasitic infections is necessary to get a complete picture of helminth co-infections. |  |
| V3(08-06-2018) | Allow inclusion of female (in additional to male) subjects. In fact the original reason to exclude women from the study was justified by the concern that women could get pregnant during the course of study, knowing that albendazole is contraindicated during pregnancy. However, the screening results from BuDiLoLo show that almost all women with high *Loa loa* parasitemia are beyond child bearing age.  -Clarify that analysis of biological safety parameters will be performed at the end of follow-up without any clinical indication.  -Clarify that Saponin method will be used to quantify Loa Loa microfilareamia only in sample that are tick smear will negative | Rella Zoleko Manego |
|  |  |  |
|  |  |  |

# Table of Contents

[Document history 2](#_Toc495523910)

[Table of Contents 3](#_Toc495523911)

[1. Summary & résumé 6](#_Toc495523912)

[1.1 English summary 6](#_Toc495523913)

[1.2 Résumé français 6](#_Toc495523914)

[2. Abbreviations 7](#_Toc495523915)

[3. Background 8](#_Toc495523916)

[3.1 Loiasis 8](#_Toc495523917)

[3.1.1 Etiology and Epidemiology 8](#_Toc495523918)

[3.1.2 Clinical aspects 8](#_Toc495523919)

[3.2 Treatment options for loiasis 9](#_Toc495523920)

[3.2.1 Diethylcarbamazine (DEC) 9](#_Toc495523921)

[3.2.2 Ivermectin (IVM) 9](#_Toc495523922)

[3.2.3 Albendazole (ALB) 10](#_Toc495523923)

[3.3 Immunology of Loiasis – a double-edged sword 10](#_Toc495523924)

[3.3.1 Immunomodulation 10](#_Toc495523925)

[3.3.2 Inflammatory responses 10](#_Toc495523926)

[4. Trial design 13](#_Toc495523927)

[4.1 Trial overview 13](#_Toc495523928)

[4.2 Objectives 14](#_Toc495523929)

[4.2.1 Primary objective 14](#_Toc495523930)

[4.2.2 Secondary objectives 14](#_Toc495523931)

[4.2.3 Exploratory objectives 14](#_Toc495523932)

[4.3 Endpoints 14](#_Toc495523933)

[4.3.1 Primary endpoints 14](#_Toc495523934)

[4.3.2 Secondary endpoints 14](#_Toc495523935)

[4.3.3 Exploratory endpoints 14](#_Toc495523936)

[4.4 Timeframe 15](#_Toc495523937)

[5. Study population 15](#_Toc495523938)

[5.1 Context 15](#_Toc495523939)

[5.2 Study size 15](#_Toc495523940)

[5.3 Inclusion criteria 16](#_Toc495523941)

[5.4 Exclusion criteria 16](#_Toc495523942)

[5.5 Recruitment & informed consent 16](#_Toc495523943)

[5.6 Withdrawal of participants 16](#_Toc495523944)

[6. Procedures 17](#_Toc495523945)

[6.1 Study schedules 17](#_Toc495523946)

[6.1.1 Arm 1 17](#_Toc495523947)

[6.1.2 Arm 2 17](#_Toc495523948)

[6.1.3 Arm 3 17](#_Toc495523949)

[6.1.4 Arm 4 17](#_Toc495523950)

[6.2 Study visits 18](#_Toc495523951)

[6.2.1 Screening 18](#_Toc495523952)

[6.2.1 Randomisation and start of study arms 18](#_Toc495523953)

[6.2.2 Visits during intake of albendazole 18](#_Toc495523954)

[6.2.3 Visits following intake of ivermectin 19](#_Toc495523955)

[6.2.4 Follow-up visits 19](#_Toc495523956)

[6.3 Treatment 19](#_Toc495523957)

[6.3.1 Albendazole 19](#_Toc495523958)

[6.3.2 Ivermectin 20](#_Toc495523959)

[6.3.3 Other 20](#_Toc495523960)

[6.4 Laboratory assessments 20](#_Toc495523961)

[6.4.1 Detection & quantification of microfilariaemia 20](#_Toc495523962)

[6.4.2 Safety parameters 20](#_Toc495523963)

[6.5 Immunology 20](#_Toc495523964)

[7. Safety 21](#_Toc495523965)

[7.1 Risks 21](#_Toc495523966)

[7.1.1 Anti-filarial treatment 21](#_Toc495523967)

[7.1.2 Phlebotomy 21](#_Toc495523968)

[7.2 Adverse events 21](#_Toc495523969)

[7.3 Solicited AEs 21](#_Toc495523970)

[7.4 Grading of AEs 21](#_Toc495523971)

[7.5 Assessment of AE relatedness 22](#_Toc495523972)

[7.6 SAEs 22](#_Toc495523973)

[7.7 Oversight 23](#_Toc495523974)

[8. Ethics 24](#_Toc495523975)

[8.1 Governing principles 24](#_Toc495523976)

[8.2 Informed consent 24](#_Toc495523977)

[8.3 Risks and benefits to study subjects 24](#_Toc495523978)

[8.4 Ethical review 24](#_Toc495523979)

[9. Data and Quality issues 24](#_Toc495523980)

[9.1 Data management 24](#_Toc495523981)

[9.2 Quality control and assurance 24](#_Toc495523982)

[10. References 26](#_Toc495523983)

# Summary & résumé

## English summary

Treatment options for loiasis, a filarial disease caused by infection with *Loa loa,* are currently limited by the very few drugs of any known efficacy and the risk of severe adverse reactions in patients with high microfilarial loads. Optimisation of regimens for the safe reduction of *L. loa* microfilaria could contribute to reduction of disease transmission, as well as facilitating elimination campaigns for lymphatic filariasis and onchocerciasis in co-endemic areas. This randomised controlled open-label pilot study will explore the efficacy, sustainability and safety of three albendazole-based treatment regimens for reducing *L. loa* microfilaraemia in infected subjects in an endemic region of Gabon.

Adult subjects of either sex with *L. loa* microfilarial loads <50,000 mf/mL identified within the ongoing loiasis epidemiology study (BuDiLoLo) in Tsamba-Magotsi department will be eligible for inclusion following provision of written informed consent. Once all (n=42) subjects are recruited, they will be randomised to one of four treatment arms: Arm 1 (n=6) will serve as control, receiving no treatment (=current standard of care); Arm 2 (n=12) will receive a three-week course of albendazole (400mg bid); Arm 3 (n=12) will receive a similar three-week course of albendazole, followed by a second (two-week) course of albendazole at the same time that subjects in Arm 4 are re-treated; Arm 4 will also receive an initial three-week course of albendazole, followed by a single dose (150 μg/Kg) of ivermectin as soon as microfilarial loads have dropped to <4,000 mf/mL in >90% of subjects. All subjects will be followed-up intensely during the treatment phase(s) for safety and measurement of microfilarial loads and thereafter every 1-2 weeks up to 6 months. The primary endpoint will be the proportion of subjects who achieve *L. loa* microfilaraemia of <100 mf/mL at 6 months. Secondary endpoints include safety & tolerability and speed/duration of microfilaricidal effects.

## Résumé français

Les options de traitement de la loiasis, une maladie filaire causée par une infection par *Loa loa*, sont actuellement limitées par de très rares médicaments d'une efficacité connue et le risque d'evenements indésirables sévères chez les patients présentant des charges microfiliaires élevées. L'optimisation des régimes pour la réduction sécuritaire de *L. loa* microfilaria pourrait contribuer à la réduction de la transmission de la maladie, ainsi qu'à faciliter les campagnes d'élimination de la filariose lymphatique et de l'onchocercose dans les zones co-endémiques. Cette étude pilote randomisée contrôlée explorera l'efficacité, la durabilité et la sécurité de trois schémas de traitement basés sur l'albendazole pour réduire la microfilarémie de *L. loa* chez des participants infectés dans une région endémique du Gabon.

Des participants des deux sexes adultes avec des charges microfiliaires de *L. loa* <50.000 mf/mL identifiés dans l'étude d'épidémiologie de loiasis (BuDiLoLo) en cours dans le département de Tsamba-Magotsi seront admissibles à l'inclusion suite à la fourniture d'un consentement éclairé écrit. Une fois que tous les participants (n = 42) sont recrutés, ils seront randomisés dans l'un des quatre bras de traitement: le Bras 1 (n = 6) servira de témoin, ne recevant aucun traitement (= niveau actuel de soins); le Bras 2 (n = 12) recevra un cours d'albendazole de trois semaines (400mg deux fois par jour); le Bras 3 (n = 12) recevra un cours similaire d'albendazole de trois semaines, suivi d'un deuxième cours d'albendazole (deux semaines) en même temps que les sujets du Bras 4 sont traités de nouveau; le Bras 4 recevra également un cours initial de trois semaines d'albendazole, suivi d'une dose unique d'ivermectine (150 μg/Kg) dès que les charges microfiliaires ont chuté à <4 000 mf/mL dans >90% des sujets. Tous les participants seront suivis intensément pendant la (les) phase(s) de traitement pour la sécurité et la mesure des charges microfiliaires et ensuite toutes les 1-2 semaines jusqu'à 6 mois. Le critère d'évaluation principal sera la proportion de participants qui atteignent une microfilarémie de *L. loa* <100 mf/mL à 6 mois. Les paramètres secondaires incluent la sécurité & la tolérance et la vitesse/durée des effets microfilaricides.

# Abbreviations

AE Adverse Event

ALB Albendazole

CEI Comité d’Ethique institutionel

CERMEL Centre de Recherches Médicales de Lambaréné

CRF Case Report Form

DEC Diethylcarbamazine

GCP Good Clinical Practice

GCLP Good Clinical Laboratory Practice

IRB Institutional Review Board

IVM Ivermectin

LF lymphatic filariasis

MDA Mass Drug Administration

mf (*L. loa*) microfilariae

OHRP Registration Office for Human Research Protections

SAE Serious Adverse Event

SOP Standard Operating Procedure

WHO World Health Organization

# Background

## Loiasis

### Etiology and Epidemiology

Loiasis, or infection caused by the filarial worm *Loa loa*, is acquired through the diurnal bite of a tabanid fly, *Chrysops dimidiata* or *C. silacea*. Adult worms live under the skin or in the intermuscular fascia and release microfilariae (mf) into the blood, with a peak around midday.

Around 30 million people live within the Loa-endemic areas of west or central Africa and 13 million are estimated to be infected ([1](#_ENREF_1), [2](#_ENREF_2)). Loa- endemic countries include: Angola, Cameroon, Central African Republic, Chad, Democratic Republic of the Congo, Equatorial Guinea, Gabon, Nigeria, Republic of the Congo, and Sudan. The overall prevalence of *L. loa* microfilaraemia in Gabon is 22.4%, with considerable regional variation ([3](#_ENREF_3)). Overall prevalence of infection is probably (much) higher, since as few as 30% of individuals infected with adult *L. loa* worms may have detectable microfilaraemia.

Although loiasis is often regarded as a relatively benign disease, it was reported to be the second or third most common cause of medical consultations in some endemic regions ([3](#_ENREF_3), [4](#_ENREF_4)).

### Clinical aspects

*L. loa* infection may present with several distinct clinical patterns ranging from asymptomatic infection to severe disease. From a parasitological point of view loiasis can be classified in occult loiasis – in the absence of microfilaraemia – and loiasis with peripheral microfilaria. The infection most commonly causes chronic skin itching, arthralgia, transient localized angioedema (callabar swelling) and sometimes an adult worm can be seen traversing the conjunctiva of an infected individual. Importantly, most of these *L. loa* associated symptoms and signs appear to be related to the adult worm and not to the microfilaria. Very rarely, infected individuals can develop renal and cardiac problems, presumably in part immune-mediated. Chronic eosinophilia in infected individuals has been associated with endo-myocardial fibrosis and related heart failure ([5](#_ENREF_5)), although in general (transient) eosinophilia is more often observed in travellers with incidental exposure than in endemic populations ([6](#_ENREF_6), [7](#_ENREF_7)). Spontaneous encephalitis has been described in some heavily infected patients ([8](#_ENREF_8)).

However, because for the vast majority of infected people symptoms are considered as benign and due to potentially life-threatening complications of inappropriate treatment, infected individuals usually do not receive curative treatment in regions of high endemicity.

Due to its globally-limited geographic distribution, the fact that it mainly affects poor rural populations and that *Loa loa* is not (even) included on the WHO’s list of neglected tropical diseases, relatively little is still known about this disease. Indeed, despite its endemic presence in Africa and the magnitude of the population infected or at risk of the disease, loiasis drew little medical, scientific or political attention until the implementation of mass drug administration (MDA) for lymphatic filariasis (LF) and onchocerciasis in the region and the associated risks experienced by *L. loa*-infected individuals became apparent (see Treatment options, below).

## Treatment options for loiasis

Curative treatment of loiasis is most commonly attempted in travellers returning after incidental exposure or in chronically-exposed patients who have migrated to non-endemic regions. Patients residing in highly endemic regions often only receive symptomatic treatment for alleviation of signs and symptoms of loiasis, due to concerns over treatment-associated adverse events and of an unfavourable risk-benefit ratio in settings with a high risk for reinfection.

Drugs active against *L. loa* are basically the same as those used for treatment of LF and onchocerciasis (e.g. in the context of MDA), see below. Since treatment-associated adverse events with these drugs are thought to be due to their rapid killing of high numbers of *L. loa* microfilariae in peripheral blood, tailored treatment regimens aimed at safely reducing *L. loa* microfilaraemia have been evaluated in small studies with the primary goal of facilitating subsequent implementation of onchocerciasis control programs – rather than of curing loiasis itself. Interestingly, no drug regimens have been evaluated so far to control and reduce the transmission of loiasis in endemic regions. Irrespective of the indication for treatment (radical cure, safe reduction of microfilaraemia or control of loiasis transmission), only very few drugs are known to be active against loiasis, targeting either the adult (macrofilaricidal) or microfilarial (microfilaricidal) developmental stages of *L. loa*. Of note, due to the absence of obligate *Wolbachia* symbionts, doxycycline treatment is not effective in *L. loa* infections.

### Diethylcarbamazine (DEC)

The drug of choice for the curative treatment of loiasis is [diethylcarbamazine](javascript:OpenWindow('./drugpopup/Diethylcarbamazine.htm','diethylcarbamazine','width=780,height=400,scrollbars=1,toolbar=yes')) (DEC) at a dose of 8-10 mg/kg per day given orally in three divided doses, although dose finding studies in *Loa loa* infection have never been performed ([9](#_ENREF_9)). The mechanism of action of DEC against filarial parasites is unclear, but appears to require an intact host immune response for full effect. Although the drug is well-tolerated in persons with low or undetectable levels of circulating microfilariae, severe adverse reactions, including renal failure and fatal encephalopathy, have been observed in the setting of high levels of microfilaraemia despite slow dose escalation and concomitant administration of steroids ([10](#_ENREF_10)). Nevertheless, such treatment regimens have been advocated for the treatment of loiasis. Overall efficacy of a three-week DEC treatment course appears to range between 30 and 60% ([11](#_ENREF_11)). Other disadvantages of DEC therapy for loiasis include the need for multiple 3-week courses in a majority of patients, and the apparent lack of efficacy in a small proportion of patients. Consequently, alternative therapies have been actively sought.

### Ivermectin (IVM)

Ivermectin, the drug of choice for the control of onchocerciasis, is a semisynthetic macrolide that is effective against the microfilariae (but not the adult worms) of *Loa loa*. A single dose of 200 mg/kg has been shown to reduce microfilarial levels by 70-90% for up to 1 year ([12](#_ENREF_12), [13](#_ENREF_13)). Resolution of the signs and symptoms of amicrofilaraemic loiasis for up to 1 year following ivermectin treatment has also been reported in two small studies ([14](#_ENREF_14)). The rapid and massive effect of ivermectin on *Loa loa* microfilariae can also lead to severe adverse drug reactions such as fatal encephalopathy in patients with a high microfilarial load ([15](#_ENREF_15)). Although low dose ivermectin therapy did appear to clear microfilaraemia less efficiently than standard dose therapy in a randomized controlled trial, the difference was not sufficient to dependably decrease the risk of ivermectin treatment in patients with high levels of microfilaraemia ([16](#_ENREF_16)). Based on these therapeutic features, safety concerns and the lack of efficacy of ivermectin against adult worms, ivermectin cannot be recommended either as curative treatment for loiasis or as a control tool in patients with high levels of microfilaraemia (>30,000 mf/mL). Whether ivermectin therapy may be administered in patients with 8000-30,000 mf/mL prior to radical cure with DEC remains controversial, as there have been no studies comparing the incidence and intensity of adverse events following DEC and ivermectin in patients with loiasis.

### Albendazole (ALB)

Albendazole – a benzimidazole anthelminthic drug - has a broader spectrum of activity and increased oral bioavailability as compared to mebendazole. In a double-blind, placebo-controlled trial ([17](#_ENREF_17)), albendazole at a dose of 400 mg per day for 21 days led to a gradual reduction of microfilarial levels in patients with loiasis to 20% of pre-treatment levels at 6 months. The slow decline in microfilaraemia is consistent with a primary effect on the adult parasites, as has been proposed in other filarial infections. Despite extremely high microfilarial levels in some patients (>50,000 mf/mL of blood), there were no clinical adverse effects and no observed hepatotoxicity, renal toxicity or hematologic abnormalities attributable to the drug. The utility of albendazole in safely reducing the numbers of circulating microfilariae (and presumably the side effects of therapy) prior to definitive treatment with DEC or ivermectin has not yet been examined. Shorter courses of albendazole (1 or 3 days) have been shown to significantly lower microfilarial counts in some studies, although the effect is variable and transient ([18](#_ENREF_18), [19](#_ENREF_19)). Albendazole has also been successfully used in the treatment of 3 patients with symptomatic loiasis refractory to multiple courses of DEC ([20](#_ENREF_20)), and should be considered in the treatment of patients refractory to or intolerant of DEC.

In summary, albendazole appears as a safe option for the treatment of loiasis irrespective of microfilaraemia level. However, appropriate regimens and the sustainability of albendazole’s effect remain to be described appropriately.

## Immunology of Loiasis – a double-edged sword

### Immunomodulation

The modulation by *L. loa* of the host immune response, which allows life-long, often asymptomatic carriage of both adult worms within host tissue and microfilariae in the blood, represents a (nevertheless still poorly understood) paragon of immunological tolerance. Indeed, Loa-specific lymphocyte proliferative responses are suppressed in chronically microfilaraemic residents of Loa-endemic areas ([21](#_ENREF_21)). In contrast, temporary visitors to Loa-endemic areas who acquire (low-grade) infection, tend to exhibit a more inflammatory eosinophil-dominated response ([6](#_ENREF_6), [7](#_ENREF_7)).

### Inflammatory responses

The other edge of Loa’s immunological ‘sword’ is the severe inflammatory reaction, especially encephalopathy, seen in a subset of Loiasis patients who are treated with diethylcarbamazine or with ivermectin in the context of MDA programmes for lymphatic filariasis or onchocerciasis, presumably due to high numbers of rapidly dying Loa microfilaria. Although almost exclusively observed in subjects with high (>30,000 mf/mL) and possibly moderate (8,000-30,0000 mf/mL) microfilarial loads, it still only occurs in a sub-set of such patients and little is understood about the considerable inter-individual risk thereof, or the underlying immunological pathways involved. Only a single case report, from our centre, has investigated the immune response in a Loiasis patient undergoing treatment, observing a transient up-regulation of lymphocyte activation markers (e.g. HLA-DR, CD69) associated with Th1-type cytokine production, followed rapidly by an immunoregulatory response ([22](#_ENREF_22)). Observations in patients undergoing treatment for lymphatic filariasis moreover suggest that clearance of microfilaria can also be associated with marked transient increase in eosinophil numbers and activation ([23](#_ENREF_23)).

**Table 1. Summary of drugs available for the treatment of *L. loa***

|  | **Regimen** | **Macrofilaricidal effect** | **Microfilaricidal effect** | **Adverse Effects** | **Contraindications** | **Comment** |
| --- | --- | --- | --- | --- | --- | --- |
| diethylcarbamazine | 10mg/kg in three divided doses for 21 days | yes | yes (direct?) | Mazotti reaction, encephalopathy in high microfilaraemia (>2500 mf/µl) | cardiac or renal impairment | Drug of choice for curative treatment with sub-optimal efficacy and problematic safety |
| ivermectin | 150µg/kg-400µg/kg single dose | no | yes (direct) | Mazotti reaction in oncho-cerciasis; encephalopathy in patients with high *L. loa* microfilaraemic loads | high *L. loa* microfilaraemia | Not useful for curative treatment of loiasis but potentially for control of transmission |
| albendazole | 200mg qd to 400mg bid for varying duration | yes | no (only indirect reduction) | minor | pregnancy | Safe drug with potential use for curative treatment and control of transmission |

**Figure 1. Overview of the trial design**


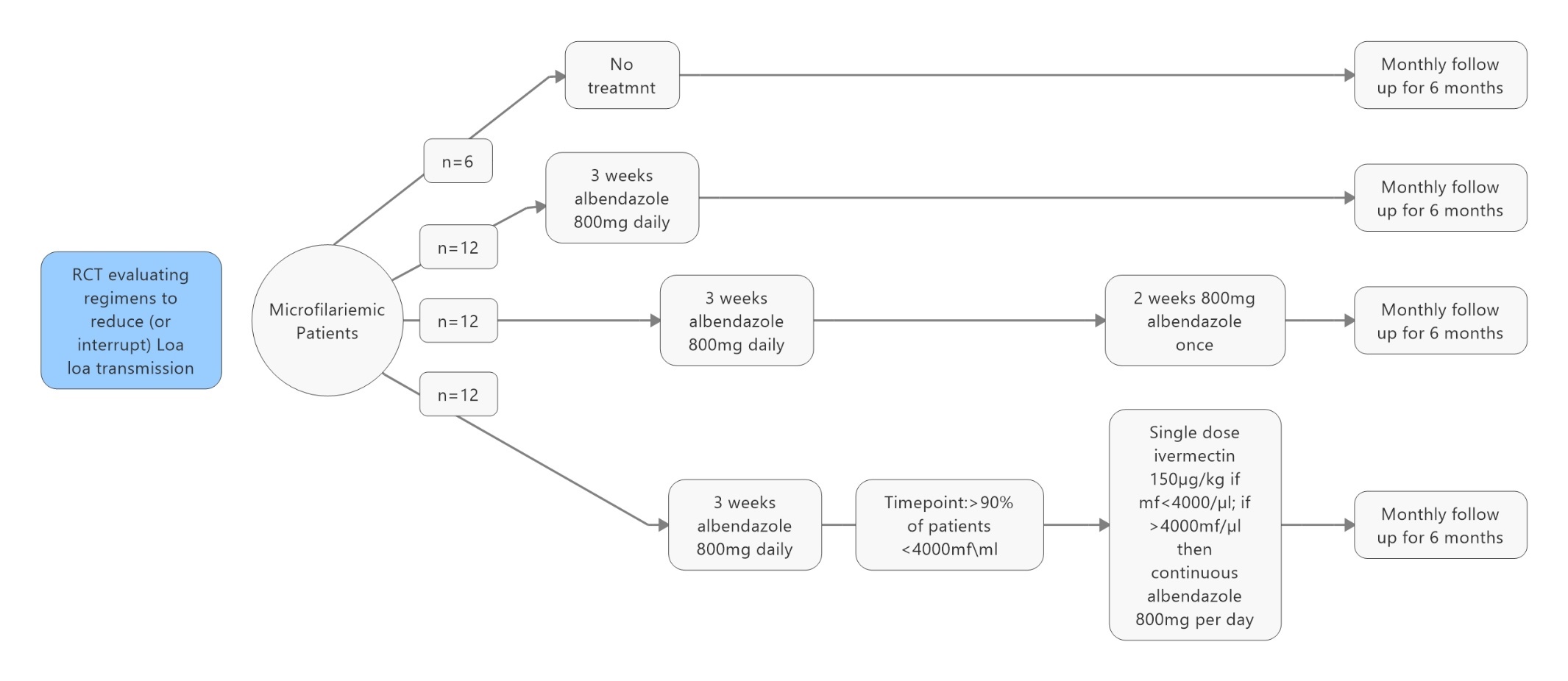


# Trial design

The overall rationale for this trial is to assess the efficacy and sustainability of available drugs in the reduction of microfilaremia in *L. loa*-infected patients in a highly endemic region of Gabon. This pilot study to be performed at the Centre de Recherches Médicales de Lambaréné (CERMEL), is designed to identify promising drug regimens for further evaluation in adequately powered comparative trials. Study outcomes will thus consist of a descriptive analysis aiming to provide the required information for the further development and evaluation of appropriate drug regimens.

## Trial overview

The study is designed as a randomised controlled open-label clinical trial to assess the safety and efficacy of different albendazole-based treatment regimens to reduce microfilaraemia in *Loa loa*-infected subjects in an endemic area of Gabon. Potential participants will be selected amongst subjects screened within the ongoing BuDiLoLo study on the epidemiology and burden of disease of *L. loa* in the Tsamba-Magotsi Department, Gabon. Adult male subjects with *L. loa* microfilaraemia < 50,000 mf/mL will be approached for inclusion into the clinical trial. Once the required number of subjects (n=42) has been recruited after signing written informed consent, all participants will be randomised into one of four study arms:

1. Control arm (n=6)
2. Single treatment course of albendazole (n=12)
3. Treatment course of albendazole followed by second course of albendazole (n=12)
4. Treatment course of albendazole followed by single dose of ivermectin (n=12)

Study arms will proceed semi-sequentially, such that arms 1 and 2 start simultaneously. After the start of arms 1+2, arms 3+4 will also commence in parallel. Subjects in arm 1 will form the control group and receive no treatment; they will be followed-up according to the same schedule as subjects in the other three treatment arms in order to evaluate natural variation in microfilaraemia with time. It is important to note in this regard that current national and international (WHO) standards of care do not indicate treatment of *L. loa* infections in endemic populations. Subjects in arm 2 will receive a single 3-week course of albendazole (400mg bid). Subjects in arm 3 will receive an identical course of albendazole, followed by a second 2-week course of albendazole at the same time when the second intervention is administered in arm 4. Subjects in arm 4 will also receive an initial 3-week course of albendazole, followed by a single dose of ivermectin (150 μg/Kg) once microfilarial concentrations drop below the threshold of 4000 mf/mL in more than 90% of individuals. Subjects in group 4 in whom this threshold is not achieved following their initial course of albendazole will instead receive a second, continuous regimen of albendazole until microfilaria are no longer detectable in peripheral blood.

Subjects in each arm of the study will be followed-up until 6 months after commencement. Figure 1 provides an overview of the trial design.

## Objectives

The overall objective of this study is to evaluate and compare different albendazole-based treatment regimes in *L. loa*-infected subjects from an endemic area, with an eye to reducing transmission for the control of loiasis in endemic regions and facilitating the safe implementation of Mass Drug Administration (MDA) programmes for LF and/or onchocerciasis in co-endemic regions.

### Primary objective

1. To evaluate the efficacy of three albendazole-based treatment regimes in reducing *L. loa* microfilarial loads

### Secondary objectives

1. To evaluate the safety and tolerability of these albendazole-based treatment regimes in *L. loa*-infected subjects
2. To estimate the speed and duration of the *L. loa* microfilaricidal effect of a single or double course of albendazole
3. To estimate the duration of the *L. loa* microfilaricidal effect of a single dose of ivermectin
4. To evaluate the variability of *L. loa* microfilaraemia in untreated subjects over time

### Exploratory objectives

1. To assess changes in (inflammatory) cytokine responses and eosinophil activation markers in *L. loa* microfilaraemic subjects treated with albendazole-based regimens in relation to reductions in microfilarial load

## Endpoints

### Primary endpoints

1. Primary efficacy endpoint will be the proportion of subjects with *L. loa* microfilarial levels <100 mf/mL at 6 months after the commencement of treatment
2. Co-primary efficacy endpoint will be the proportion of subjects without microscopically detectable microfilariae (assessed on three consecutive days) at any time point during follow up.

### Secondary endpoints

1. Primary safety endpoint will be the occurrence of any grade 3 adverse event (AE) or serious adverse event (SAE), from the commencement of each study arm until 6 months thereafter; such events may be related or unrelated to study procedure.
2. Safety & tolerability will additionally be assessed by the occurrence of all (grade 1-2) adverse events at least possibly related to study therapy, from the commencement of each study arm until 6 months thereafter.
3. Proportion of subjects without peripheral microfilaraemia at 6 months follow up
4. Median reduction of *L. loa* MF at each month post start of treatment and at nadir
5. Median time from commencement of treatment to nadir of *L. loa* microfilaraemia

### Exploratory endpoints

1. Immunological endpoints include circulating and *in vitro* inducible cytokine levels and markers of eosinophil activation measured at baseline, during and after albendazole/ivermectin treatment and at the end of follow-up.

## Timeframe

The trial is set to start including participants as soon as ethical clearance is obtained (e.g. by October 2017), since the underlying BuDiLoLo study has already started screening subjects. The recruitment phase ends once sufficient participants (n=42) are included for the overall design of the trial. The duration of the recruitment phase thus depends partially on the screening rate in BuDiLoLo, but is anticipated not to exceed ~2 months. Arms 1 and 2 of the trial will commence as soon as recruitment is completed and randomization is performed. Arms 3 and 4 will commence in parallel thereafter. Follow-up in each study arm will last 6 months. Thus the total duration of the trial is anticipated to be ~9-10 months.

# Study population

## Context

All study participants for this treatment trial will be recruited amongst study subjects enrolled in the ongoing Burden of Disease in *Loa loa* (BuDiLoLo) study. The aims and design of this study are described the BuDiLoLo study protocol. Briefly, the primary objective of the BuDiLoLo study is to assess the burden of disease, including DALYs lost and economic impact, caused by *Loa loa* on the local population in central Gabon. The study is cross-sectional in design and based in & around the town of Fugamou in the Tsamba-Magotsi department, Ngounié province and in the neighbouring Moyen-Ogooué province, Gabon. Ngounié is considered highly endemic for *L. loa*, with an estimated prevalence of +-18% ([3](#_ENREF_3), [24](#_ENREF_24)); in contrast, Moyen-Ogooué is considered low-endemic for loiasis. 1000-2500 subjects of all ages (≥2 years) from randomly selected households in a random sample of villages will be included with the target of obtaining ≥700 *Loa*-infected individuals. Subjects will be interviewed using the WHO-validated RAPLOA questionnaire and a single blood draw will be performed for amongst others the detection/quantification of *L. loa* and *Mansonella* spp. microfilaria by thick smear microscopy, leukocyte concentration techniques and PCR.

Adult both sex subjects with *L. loa* infection levels < 50,000 mf/mL will be considered eligible for participation in the treatment study. Once sufficient participants are included for the entire study design (see below), they will be randomized to one of four study arms, which will commence semi-sequentially as described above.

## Study size

This study is a descriptive study and is not powered for statistical testing of a predefined hypothesis. As a proof of principle study, this trial aims to describe the crude effect of respective treatment regimens on the proportion of subjects achieving sustained reduction in peripheral microfilaraemia and on the kinetics of parasitaemia. For this purpose a total of 8 evaluable subjects per treatment arm is required at the end of 6-months’ follow-up. In order to allowing for up to ⅓ (33%) loss to follow-up during this period, 12 patients will be recruited into each treatment group. An additional 6 patients will be recruited for the control group for the description of the natural variability of microfilaraemia. Thus the total study size will be 42 participants: n=6 in study arm 1 and n=12 each in arms 2-4. In case of drop out of more than 33% in any study arm for evaluation of the primary endpoint, further patients will be screened and included in the respective study arm to achieve at least 8 evaluable patients in each treatment group.

## Inclusion criteria

- Adult, participants of either sex
- *L. loa* microfilaria < 50,000 mf/mL
- Intention to remain residing in study area and comply with study procedures throughout 6 month follow-up period
- Signed written informed consent

## Exclusion criteria

- Patients treated with albendazole during the 4 previous weeks
- Known intolerance or allergy to any study drug
- Viral hepatitis (HBV, HCV) or other known active liver disease
- HIV or other known immunosuppressive condition
- History of epilepsy, encephalitis, meningitis or encephalopathy
- Any other acute or chronic medical condition, medication or psychosocial factor(s) deemed by the Investigator to put the potential participant at greater than acceptable risk or to significantly affect study outcomes
- Pregnancy, no breastfeeding and no agree to use a contraceptive method for women with childbearing potential

## Recruitment & informed consent

Adult male subjects in the BuDiLoLo study found to be harbouring *L. loa* microfilariaemia < 50,000 mf/mL will be approached by study team with regards to possible participation in the trial. The aims, design, in- & exclusion criteria and procedures of the trial will be provided to them in written form as well as explained to them in understandable terms and they will be given adequate time (≥2 days) to consider.

If following this period they are still interested in participating, potential participants will be invited for a screening visit where they will first be asked to sign a written informed consent form. In the case of illiterate participants, an impartial witness will be asked to co-sign the consent form.

## Withdrawal of participants

Participants may be withdrawn from the study prior to completion of follow-up, under the following circumstances:

- On direct request of the participant; the participant need not provide any reason for withdrawal
- If in the opinion of investigator(s), safety monitor and/or supervising ethical committee continued participation poses unacceptable risks to one or more subjects

If further follow-up of withdrawn participants is considered important for purely safety purposes, the participant(s) will be informed of this and of the risks of loss to follow-up will be clearly explained to them.

# Procedures

## Study schedules

Arms 1+2 will commence simultaneously; after arms 1+2 have started, arms 3+4 will also start in parallel. Basic follow-up schedules will be similar for subjects in all 4 arms: visits will take place at screening, immediately prior to commencement of the study arm and thereafter daily for the first week, twice per week for the subsequent two weeks (i.e. until the end of the first albendazole course in arms 2-4) and thereafter once per week until the second course of treatment commences in arms 3+4. This second round of treatment will commence as soon as 90% of the subjects still in follow-up in arm 4 drop below the threshold of 4000 mf/mL. A similar visit schedule will then be used for the second treatment round in arms 3+4 (daily during the first week of treatment and twice per week during the second week). Thereafter, twice monthly follow up visits will be scheduled for the remaining follow-up period until the end of follow-up at 6 months.

### Arm 1

No anti-helminthic medication will be used, although symptomatic treatment may be provided if deemed clinically appropriate. Participants will be seen at screening, immediately prior to commencement of the study arm and thereafter daily for the first week, twice per week for the subsequent two weeks and thereafter once per week until eight weeks after start of the study arm and then twice monthly until the end of follow-up at 6 months. At the end of the study, any participants in this arm who wish to, will be provided with a 3-week course of albendazole (400mg/day), in line with Gabonese national guidelines for MDA in Loa-endemic populations. Note that no further follow-up will be performed during this treatment course.

### Arm 2

A single 3-week course of albendazole (800mg/day) will be provided at commencement of the study arm, simultaneously with the start of Arm 1. Participants will be seen at screening, immediately prior to commencement of the study arm and thereafter daily for the first week, twice per week for the subsequent two weeks (until the end of the albendazole course) and thereafter once per week until eight weeks after start of the study arm and then twice monthly until the end of follow-up at 6 months.

### Arm 3

A single 3-week course of albendazole (800mg/day) will be provided at commencement of the study arm, simultaneously with the start of Arm 4. Participants will be seen at screening, immediately prior to commencement of the study arm and thereafter daily for the first week, twice per week for the subsequent two weeks (i.e. until the end of first albendazole course) and thereafter once per week until 90% of the subjects still in follow-up in arm 4 drop below the threshold of 4000 mf/mL; if this criterion is not yet achieved by week 8, subsequent visits will be reduced to twice monthly. All participants will then be re-treated with a 2-week course of albendazole (800mg/day). All subjects will be seen daily for follow-up visits during the first week of re-treatment and twice per week during the second week. Thereafter further follow-up visits will take place twice monthly until the end of follow-up at 6 months.

### Arm 4

A single 3-week course of albendazole (800mg/day) will be provided at commencement of the study arm, simultaneously with the start of Arm 3. Participants will be seen at screening, immediately prior to commencement of the study arm and thereafter daily for the first week, twice per week for the subsequent two weeks (i.e. until the end of first albendazole course) and thereafter once per week until 90% of the subjects still in follow-up in arm 4 drop below the threshold of 4000 mf/mL; if this criterion is not yet achieved by week 8, subsequent visits will be reduced to twice monthly. All participants with <4000 mf/mL will then be treated with a single dose of ivermectin; any subjects *not* achieving <4000 mf/mL at this time point will instead commence continuous re-treatment with *albendazole* until microfilaraemia becomes undetectable, until treatment is no longer tolerated, or until the end of follow-up (whichever occurs first). *All* subjects will be seen daily for follow-up visits during the first week following ivermectin/albendazole (re-)treatment and twice per week during the second week. Thereafter further follow-up visits will take place twice monthly until the end of follow-up at 6 months.

## Study visits

### Screening

Potential participants will be informed again about the study aims, design, in- & exclusion criteria and procedures. If still willing to participate, subjects will be asked to sign written informed consent. Thereafter, a full medical history and concomitant medication will be recorded and a physical examination will be performed. Blood will be drawn for laboratory safety parameters (haematology, biochemistry, serology) and to confirm microfilarial levels and to exclude pregnancy in female subjects. In addition a single stool sample and urine sample will be collected to assess
helminth infection (since we will be treating with albendazole which
will also have an effect on many helminths), which could affect
tolerability and influence non-specific symptoms such as pruritus. Indeed helminth infection also affect immune response, therefore it will be necessary to know the subject helminth status.

Any abnormal findings that result in exclusion from the study and/or require treatment will be communicated to the subject; for any treatment required beyond that which can be provided directly by the study team, subjects will be referred to other healthcare providers as appropriate.

### Randomisation and start of study arms

Once sufficient participants are included for the entire study, participants will be randomized to one of the four study arms. The generation of the randomization sequence will be computer assisted with a block size of 10 with a fixed allocation of 12 patients for each treatment arm and 6 for the control group. The randomisation list will be produced by a team independent of the investigators and will be provided as sealed opaque envelopes to the study team. The investigator may only open the sealed opaque envelope after informed consent has been obtained and the subject is to be allocated to a treatment arm. Treatment allocation will then be revealed to the subject. No change of treatment allocation will be permitted in the course of this trial. If it is in the interest of the patient safety, the patient will be removed from the study and will receive appropriate routine care. In case of requirement of allocation of extra-patients to a study arm due to excessive loss-to-follow-up, patients will be screened and will be allocated to the respective treatment arm without randomization. This fact will be clearly acknowledged in the analysis and presentation of the data.

At the first visit of their study arm (DT0), each participant will again have blood drawn for laboratory safety parameters (haematology and biochemistry) and for quantification of *L. loa* microfilaraemia. In addition, blood may be collected for immunology (baseline). Participants in arms 2, 3 & 4 will receive their first dose of albendazole under supervision.

### Visits during intake of albendazole

Once daily visits will be performed during the first week of all study arms, including observed intake of one daily dose of albendazole in arms 2-4 and recording of AEs in all. For the two weeks thereafter, visits will be performed twice weekly. Patients will be specifically and repeatedly advised that albendazole intake shall follow intake of a standard meal as this importantly enhances the bioavailability of albendazole. Note that intake of albendazole on non-visit days and the intake of the *second* dose on visit days will not be supervised. Blood samples will be drawn for laboratory safety parameters (haematology and biochemistry) and quantification of microfilaraemia on the 1^st^ day after start of treatment (DT+1), DT+3, DT+7, DT+10, DT+14, DT+17 and DT+21. In arm 1, blood samples will be drawn for the quantification of microfilaraemia according to a similar schedule. At DT+14 additional blood may be collected for immunology in all study arms. (*Visit designations are approximate and may vary by ±1 day in the first week after start of treatment and by ±2 days in the two weeks thereafter.*).

If clinically indicated a physical examination and/or additional blood draw(s) for laboratory safety parameters will be performed.

Subjects in arm 3 (and subjects in arm 4 who failed to achieve <4000 mf/mL) will undergo a similar schedule of visits during their second course of albendazole, commencing on the first day of re-treatment (DRT0) except that these visits will last only until DRT+14, instead of DRT+21. Additional blood may be collected for immunology at DRT0 and DRT+14. (*Visit designations are approximate and may vary by ±1 day in the first week after start of treatment and by ±2 days in the week thereafter.*)

### Visits following intake of ivermectin

Subjects in arm 4 who achieve <4,000 mf/mL will receive a single dose of ivermectin under observation, followed by daily visits to record AEs during the first week after intake and twice weekly in the second week. Blood will be drawn for laboratory safety parameters (haematology and biochemistry) and quantification of microfilaraemia on the day of treatment (DRT0), DRT+1, DRT+3, DRT+7, DRT10 and DRT+14. Additional blood may be collected for immunology at DRT0, DRT+1 and DRT+14. (*Visit designations are approximate and may vary by ±1 day in the first week after start of treatment and by ±2 days in the week thereafter.*)

If clinically indicated a physical examination and/or additional blood draw(s) for laboratory safety parameters will be performed.

### Follow-up visits

In arms 1+2, weekly follow-up visits will take place from week 4 through week 8; thereafter, twice monthly visits will take place until the end of follow-up (at month 6). In arms 3+4 weekly follow-up visits will take place between the first and second courses of treatment; if re-treatment has not started by week 8, then these visits will be reduced to twice monthly. Twice monthly visits will also take place from two weeks after commencement re-treatment until the end of follow-up (at month 6) in arms 3+4. AEs will be recorded and blood drawn for quantification of microfilaraemia at each visit. Additional blood may be collected for immunology and laboratory safety parameters at the end of follow-up (at month 6) in all study arms. If clinically indicated a physical examination and/or additional blood draw(s) for laboratory safety parameters will be performed. At day 28, day 63 and at 6 months an additional pregnancy test will be performed in female subjects.

The investigator will collect pregnancy information on female participant who becomes pregnant during drug intake or three months after the last dose of study drug. The participant will be follow up to delivery and the neonate information will also be collected. Any termination of pregnancy will be reported.

## Treatment

### Albendazole

The initial treatment course in arms 2-4 will consist of 400mg bid for three weeks. The second course in arm 3 will consist of 400mg bid for three weeks. Subjects in arm 4 who have not achieved <4000 mf/mL after their first course of albendazole will instead be put on a continuous course of 400mg bid. Albendazole will be provided as 400mg tablets. Patients will be specifically and repeatedly advised that albendazole intake shall follow intake of a standard meal as this importantly enhances the bioavailability of albendazole. Note that at the end of the study, any participants in arm 1 who wish to, will be provided with a 3-week course of albendazole (400mg/day), in line with Gabonese national guidelines for MDA in Loa-endemic populations.

### Ivermectin

Ivermectin will be taken as a stat dose of 150 μg/Kg by subjects in arm 4 who have achieved <4000 mf/mL after their first course of albendazole. Ivermectin will be provided as tablets of 3mg.

### Other

Symptomatic treatment may be provided to subjects in all study arms as clinically indicated, e.g. antihistamines for itching or paracetamol for headache and joint pain.

## Laboratory assessments

### Detection & quantification of microfilariaemia

*L. loa* microfilaraemia will be detected and quantified in peripheral venous blood using Giemsa-stained thick smear microscopy and/or the saponin concentration method as appropriate: thick smear microscopy will be performed first for all samples and saponin concentration will in principle only be performed on samples which are negative for microfilaria by thick smear. Thick smear microscopy will be performed using the Lambaréné method, using a fixed volume (10μL) of blood on a fixed slide surface area (1.8 cm^2^) and read at 10x magnification with a known microscopic optical field diameter in order to allow quantification of microfilaraemia per optical field. Unstained, unfixed sediments of 1mL of saponin-lysed whole blood will be observed at 10x magnification in their entirety in order to quantify microfilaraemia. PCR may be performed on samples as an additional detection/quantification tool, if required. Blood for parasite detection/quantification will be taken from remaining material in one or both of the tubes already collected for respectively haematology and biochemistry, see section 6.4.2. Safety parameters (below).

### Safety parameters

Laboratory safety parameters to be measured include full blood count (Hb, Ht, RBC parameters, WBCs, leuko diff, Tr) using 1.2-2.7 ml of EDTA blood and if scheduled (e.g. during treatment), or otherwise clinically indicated, a biochemistry panel (CRP, ALAT, ASAT, γGT, bilirubin, creat, electrolytes and glucose, as appropriate) using 2.7 ml of lithium-heparin blood.

## Immunology

Up to 10mL of lithium heparin blood may be drawn at each immunological assessment time-point (see section 6.2 study visits). Assessment of study subjects may include determination of leukocyte differential counts & immunophenotyping by flow cytometry and quantification of circulating cytokines, eotaxins, eosinophilic degranulation proteins and total IgE in plasma by multiplex bead array and/or ELISA. PBMC and 1:5 diluted whole blood stimulation assays may be performed with Loa-specific antigen (crude adult worm lysate and crude microfilarial lysate) and non-specific stimuli (anti-CD3/PHA) at baseline, during and after albendazole/ivermectin treatment and at the end of follow-up. A panel of Th1-, Th2- and Th17-archetype as well as anti-inflammatory cytokines may be measured in PBMC culture supernatant by multiplex bead array and the predominance & activation of individual Th cell-subsets may be determined by flow cytometry. Eosinophil degranulation proteins may be measured in supernatants of whole blood assays and a dedicated flow cytometry panel may be used to assess non-secreted markers of eosinophil activation (e.g. CD69 and MHC-II up-regulation).

# Safety

## Risks

### Anti-filarial treatment

The risk of treatment-associated adverse events in loiasis are largely associated with the drugs DEC and ivermectin and are thought to be due to their rapid killing of high numbers of *L. loa* microfilariae in peripheral blood. Treatment with albendazole has generally not been associated with such adverse reactions. By only providing ivermectin treatment to those subjects who have already achieved a reduction of microfilaraemia to below 4000 mf/mL, the associated risk of treatment is almost negligible. However, close clinical and laboratory follow-up is planned to optimise the management of any unforeseen events. Symptomatic treatment, e.g. antihistamines, paracetamol, etc., will be provided to all subjects as clinically indicated. A clinical trial insurance will be obtained to cover all patients participating in this trial.

### Phlebotomy

The total volume of venous whole blood drawn over the entire trial, over a ±6 month period, will not exceed 500mL (i.e. the standard volume of blood donation). There may be minor bruising, local tenderness or symptoms of pre-syncope associated with venepuncture, which will not be documented as AEs if they occur. In rare cases arteries or nervous tissue may be injured or a punctured vessel may occlude and induce inflammation of the surrounding tissue.

## Adverse events

An adverse event (AE) is defined as any untoward medical occurrence in a patient or clinical investigation subject administered a pharmaceutical product and which does not necessarily have a causal relationship with this treatment. An adverse event (AE) can therefore be any unfavourable and unintended sign (including an abnormal laboratory finding), symptom, or disease temporally associated with the use of a medicinal (investigational) product, whether or not related to the medicinal (investigational) product.

## Solicited AEs

No solicited AEs will be sought in this study.

## Grading of AEs

All AEs will be assessed for severity by the investigator. Inherent in this assessment is the medical and clinical consideration of all information surrounding the event including any medical intervention required. Each event will be assigned one of the following categories: mild, moderate, severe, or life-threatening. The criteria below may be used for any symptom not included in the grading scale. Any grade 4 (life-threatening) AE must be reported as an SAE.

The CRF for AEs will reflect only the highest severity for continuous days an event occurred.

• Grade 1 (Mild): Does not interfere with routine activities, minimal level of discomfort

• Grade 2 (Moderate): Interferes with routine activities, moderate level of discomfort

• Grade 3 (Severe): Unable to perform routine activities, significant level of discomfort

• Grade 4 (Potentially life-threatening): Hospitalization or ER visit for potentially

life-threatening event

If a subject is evaluated in an emergency room for nonlife threatening illness or symptoms (i.e., visits emergency department on weekend for mild problems because the physician’s office is closed), the information from that visit will be reviewed and severity of the adverse event will be assessed according to the subject’s clinical signs and symptoms.

As defined by the ICH guideline for GCP, the term “severe” is often used to describe intensity (severity) of a specific event (as in mild, moderate, or severe myocardial infarction); the event itself however, may be of relatively minor medical significance (such as severe headache). This is not the same as “serious”, which is based on subject/event outcome or action criteria usually associated with events that pose a threat to a subject’s life or functioning. Seriousness (not severity) serves as a guide for defining regulatory reporting obligations.

## Assessment of AE relatedness

The investigator must assign a relationship of each AE to the receipt of study medication. The investigator will use clinical judgment in conjunction with the assessment of a plausible biologic mechanism, a temporal relationship between the onset of the event in relation to receipt of the investigational product(s), and identification of possible alternate aetiologies including underlying disease, concurrent illness or concomitant medications. The following guidelines should be used by investigators to assess the relationship of an AE to study medication:

• Not related: No relationship to investigational product. Applies to those events for which evidence exists that there is an alternate aetiology.

• Unlikely: Likely unrelated to the investigational product. Likely to be related to factors other than investigational product, but cannot be ruled out with certainty.

• Possible: An association between the event and the administration of investigational product cannot be ruled out. There is a reasonable temporal association, but there may also be an alternative etiology such as the subject’s clinical status or underlying factors including other therapy.

• Probable: There is a high degree of certainty that a relationship to the investigational product exists. There is a reasonable temporal association, and the event cannot be explained by known characteristics of the subject’s clinical state or factors including other therapy.

• Definite: An association exists between the receipt of investigational product and the event. An association to other factors has been ruled out

## SAEs

An AE is considered “serious” if it results in any of the following outcomes:

• Death

• A life-threatening situation

• Inpatient hospitalization or prolongation of existing hospitalization

• Persistent or significant disability/incapacity defined as a “substantial disruption of the ability to conduct normal life functions”

• Congenital anomaly/birth defect (in the offspring of a subject)

An AE or suspected adverse reaction is considered “life-threatening” if its occurrence places the patient or subject at immediate risk of death. It does not include an adverse event or suspected adverse reaction that, had it occurred in a more severe form, might have caused death.

Important medical events that may not result in death, be life-threatening, or require hospitalization may be considered serious when, based upon appropriate medical judgment, they may jeopardize the patient or subject and may require medical or surgical intervention to prevent one of the outcomes listed in this definition. Examples of such medical events include allergic bronchospasm requiring intensive treatment in an emergency room or at home, blood dyscrasias or convulsions that do not result in inpatient hospitalization, or the development of drug dependency or drug abuse.

SAEs must be reported to the safety monitor and responsible ethical committee within 24 hours.

SAEs will be collected and recorded from all participants throughout the trial, from the moment of inclusion until the end of follow-up (i.e. 6 month after commencement of each study arm).

## Oversight

A safety monitor, i.e. a clinician with experience in clinical trials who is permanently on site and is independent of the clinical trial team, will oversee participant safety. He/she may recommend halting or stopping the trial.

# Ethics

## Governing principles

This study will be performed in accordance with the current revision of the Declaration of Helsinki and the latest versions of the ICH guidelines for Good Clinical Practice (GCP) and guidelines for Good Clinical Laboratory Practice (GCLP).

## Informed consent

Written, informed consent will be obtained from all study subjects prior to inclusion, as described above. This consent may be withdrawn by the study subject at any time, without being required to provide a reason.

## Risks and benefits to study subjects

Risks to study subjects are discussed in section 7.1 Risks. Benefits include provision of symptomatic treatment for signs and symptoms of loiasis to subjects in all arms throughout the study. Note that the anti-filarial treatment provided to subjects in arms 2-4 cannot be defined as a benefit, since the (long-term) parasitological and clinical efficacy of albendazole and ivermectin in Loa-endemic populations remains to be clarified.

## Ethical review

This trial will be submitted for ethical review to the Institutional Review Board (Comité d’Ethique institutionel, CEI) of the Centre de Recherches Médicales de Lambaréné (CERMEL), which is registered with the Registration Office for Human Research Protections (OHRP) with the numbers: IORG0007336 / IRB00008812.

# Data and Quality issues

## Data management

All study subjects will allocated a unique pseudonymised study code at screening; only investigators, safety monitor, IRB and Sponsor will have access to these records. Data will be recorded on paper case record forms (CRFs) using these study codes and will be entered in an electronic database for further analysis. All laboratory samples will also be labelled, processed and stored until end of analysis but no longer than 5 years using these study codes. Data analysis will be performed following a predefined statistical analysis plan.

## Quality control and assurance

All study procedures, whether clinical or laboratory-based will be performed in accordance with the current versions of CERMEL’s relevant Standard Operation Procedures (SOPs). CERMEL also has an overall Quality Assurance programme in operation. An external monitor will perform regular monitoring to check that the trial is conducted, and that data are recorded, analysed & accurately reported, according to study protocols, relevant SOPs and in compliance with ICH GCP. Internal audits are also performed of laboratory activities and in addition CERMEL’s laboratories are enrolled in External Quality Assurance programmes.

# References

1. Chesnais CB, Takougang I, Paguele M, Pion SD, Boussinesq M. Excess mortality associated with loiasis: a retrospective population-based cohort study. The Lancet Infectious diseases. 2017;17(1):108-16.

2. Zoure HG, Wanji S, Noma M, Amazigo UV, Diggle PJ, Tekle AH, et al. The geographic distribution of Loa loa in Africa: results of large-scale implementation of the Rapid Assessment Procedure for Loiasis (RAPLOA). PLoS neglected tropical diseases. 2011;5(6):e1210.

3. Akue JP, Nkoghe D, Padilla C, Moussavou G, Moukana H, Mbou RA, et al. Epidemiology of concomitant infection due to Loa loa and Mansonella perstans in Gabon. PLoS neglected tropical diseases. 2011;5(10):e1329.

4. Metzger WG, Mordmuller B. Loa loa-does it deserve to be neglected? The Lancet Infectious diseases. 2014;14(4):353-7.

5. Andy JJ, Bishara FF, Soyinka OO, Odesanmi WO. Loasis as a possible trigger of African endomyocardial fibrosis: a case report from Nigeria. Acta Trop. 1981;38(2):179-86.

6. Herrick JA, Metenou S, Makiya MA, Taylar-Williams CA, Law MA, Klion AD, et al. Eosinophil-associated processes underlie differences in clinical presentation of loiasis between temporary residents and those indigenous to Loa-endemic areas. Clinical infectious diseases : an official publication of the Infectious Diseases Society of America. 2015;60(1):55-63.

7. Klion AD, Massougbodji A, Sadeler BC, Ottesen EA, Nutman TB. Loiasis in endemic and nonendemic populations: immunologically mediated differences in clinical presentation. J Infect Dis. 1991;163(6):1318-25.

8. Lukiana T, Mandina M, Situakibanza NH, Mbula MM, Lepira BF, Odio WT, et al. A possible case of spontaneous Loa loa encephalopathy associated with a glomerulopathy. Filaria J. 2006;5:6.

9. Drugs for parasitic infections. Med Lett Drugs Ther. 1998;40(1017):1-12.

10. Carme B, Boulesteix J, Boutes H, Puruehnce MF. Five cases of encephalitis during treatment of loiasis with diethylcarbamazine. Am J Trop Med Hyg. 1991;44(6):684-90.

11. Wahl G, Georges AJ. Current knowledge on the epidemiology, diagnosis, immunology, and treatment of loiasis. Trop Med Parasitol. 1995;46(4):287-91.

12. Gardon J, Kamgno J, Folefack G, Gardon-Wendel N, Bouchite B, Boussinesq M. Marked decrease in Loa loa microfilaraemia six and twelve months after a single dose of ivermectin. Trans R Soc Trop Med Hyg. 1997;91(5):593-4.

13. Martin-Prevel Y, Cosnefroy JY, Tshipamba P, Ngari P, Chodakewitz JA, Pinder M. Tolerance and efficacy of single high-dose ivermectin for the treatment of loiasis. Am J Trop Med Hyg. 1993;48(2):186-92.

14. Gantois N, Rapp C, Gautret P, Ficko C, Savini H, Larreche S, et al. Imported loiasis in France: a retrospective analysis of 47 cases. Travel medicine and infectious disease. 2013;11(6):366-73.

15. Boussinesq M, Gardon J, Gardon-Wendel N, Chippaux JP. Clinical picture, epidemiology and outcome of Loa-associated serious adverse events related to mass ivermectin treatment of onchocerciasis in Cameroon. Filaria J. 2003;2 Suppl 1:S4.

16. Kamgno J, Pion SD, Tejiokem MC, Twum-Danso NA, Thylefors B, Boussinesq M. Randomized, controlled, double-blind trial with ivermectin on Loa loa microfilaraemia: efficacy of a low dose (approximately 25 microg/kg) versus current standard dose (150 microg/kg). Trans R Soc Trop Med Hyg. 2007;101(8):777-85.

17. Klion AD, Massougbodji A, Horton J, Ekoue S, Lanmasso T, Ahouissou NL, et al. Albendazole in human loiasis: results of a double-blind, placebo-controlled trial. J Infect Dis. 1993;168(1):202-6.

18. Tabi TE, Befidi-Mengue R, Nutman TB, Horton J, Folefack A, Pensia E, et al. Human loiasis in a Cameroonian village: a double-blind, placebo-controlled, crossover clinical trial of a three-day albendazole regimen. Am J Trop Med Hyg. 2004;71(2):211-5.

19. Tsague-Dongmo L, Kamgno J, Pion SD, Moyou-Somo R, Boussinesq M. Effects of a 3-day regimen of albendazole (800 mg daily) on Loa loa microfilaraemia. Annals of tropical medicine and parasitology. 2002;96(7):707-15.

20. Klion AD, Horton J, Nutman TB. Albendazole therapy for loiasis refractory to diethylcarbamazine treatment. Clinical infectious diseases : an official publication of the Infectious Diseases Society of America. 1999;29(3):680-2.

21. Baize S, Wahl G, Soboslay PT, Egwang TG, Georges AJ. T helper responsiveness in human Loa loa infection; defective specific proliferation and cytokine production by CD4+ T cells from microfilaraemic subjects compared with amicrofilaraemics. Clin Exp Immunol. 1997;108(2):272-8.

22. Winkler S, Paiha S, Winkler H, Graninger W, Marberger M, Steiner GE. Microfilarial clearance in loiasis involves elevation of Th1 and Th2 products and emergence of a specific pattern of T-cell populations. Parasite Immunol. 1996;18(9):479-82.

23. Gopinath R, Hanna LE, Kumaraswami V, Perumal V, Kavitha V, Vijayasekaran V, et al. Perturbations in eosinophil homeostasis following treatment of lymphatic filariasis. Infect Immun. 2000;68(1):93-9.

24. Mombo-Ngoma G, Mackanga JR, Basra A, Capan M, Manego RZ, Adegnika AA, et al. Loa loa Infection in Pregnant Women, Gabon. Emerg Infect Dis. 2015;21(5):899a-901.
